# Loss of stability and unfolding cooperativity in hPGK1 upon gradual structural perturbation of its N-terminal domain hydrophobic core

**DOI:** 10.1038/s41598-022-22088-1

**Published:** 2022-10-13

**Authors:** Juan Luis Pacheco-García, Dmitry S. Loginov, Athi N. Naganathan, Pavla Vankova, Mario Cano-Muñoz, Petr Man, Angel L. Pey

**Affiliations:** 1 Departamento de Química Física, Facultad de Ciencias, Universidad de Granada, Av. Fuentenueva s/n, 18071 Granada, Spain; 2 Institute of Microbiology of the Czech Academy of Sciences, BioCeV, Prumyslova 595, 252 50 Vestec, Czech Republic; 3 Department of Biotechnology, Bhupat & Jyoti Mehta School of Biosciences, Indian Institute of Technology Madras (IITM), Chennai, 600036 India; 4 Institute of Biotechnology of the Czech Academy of Sciences, BioCeV, Prumyslova 595, 252 50 Vestec, Czech Republic; 5 Departamento de Química Física, Unidad de Excelencia de Química aplicada a Biomedicina y Medioambiente e Instituto de Biotecnología, Facultad de Ciencias, Universidad de Granada, Av. Fuentenueva s/n, 18071 Granada, Spain

**Keywords:** Biophysical chemistry, Protein folding, Proteins, Structural biology, Biophysics, Structural biology

## Abstract

Phosphoglycerate kinase has been a model for the stability, folding cooperativity and catalysis of a two-domain protein. The human isoform 1 (hPGK1) is associated with cancer development and rare genetic diseases that affect several of its features. To investigate how mutations affect hPGK1 folding landscape and interaction networks, we have introduced mutations at a buried site in the N-terminal domain (F25 mutants) that either created cavities (F25L, F25V, F25A), enhanced conformational entropy (F25G) or introduced structural strain (F25W) and evaluated their effects using biophysical experimental and theoretical methods. All F25 mutants folded well, but showed reduced unfolding cooperativity, kinetic stability and altered activation energetics according to the results from thermal and chemical denaturation analyses. These alterations correlated well with the structural perturbation caused by mutations in the N-terminal domain and the destabilization caused in the interdomain interface as revealed by H/D exchange under native conditions. Importantly, experimental and theoretical analyses showed that these effects are significant even when the perturbation is mild and local. Our approach will be useful to establish the molecular basis of hPGK1 genotype–phenotype correlations due to phosphorylation events and single amino acid substitutions associated with disease.

## Introduction

**Figure 1:**
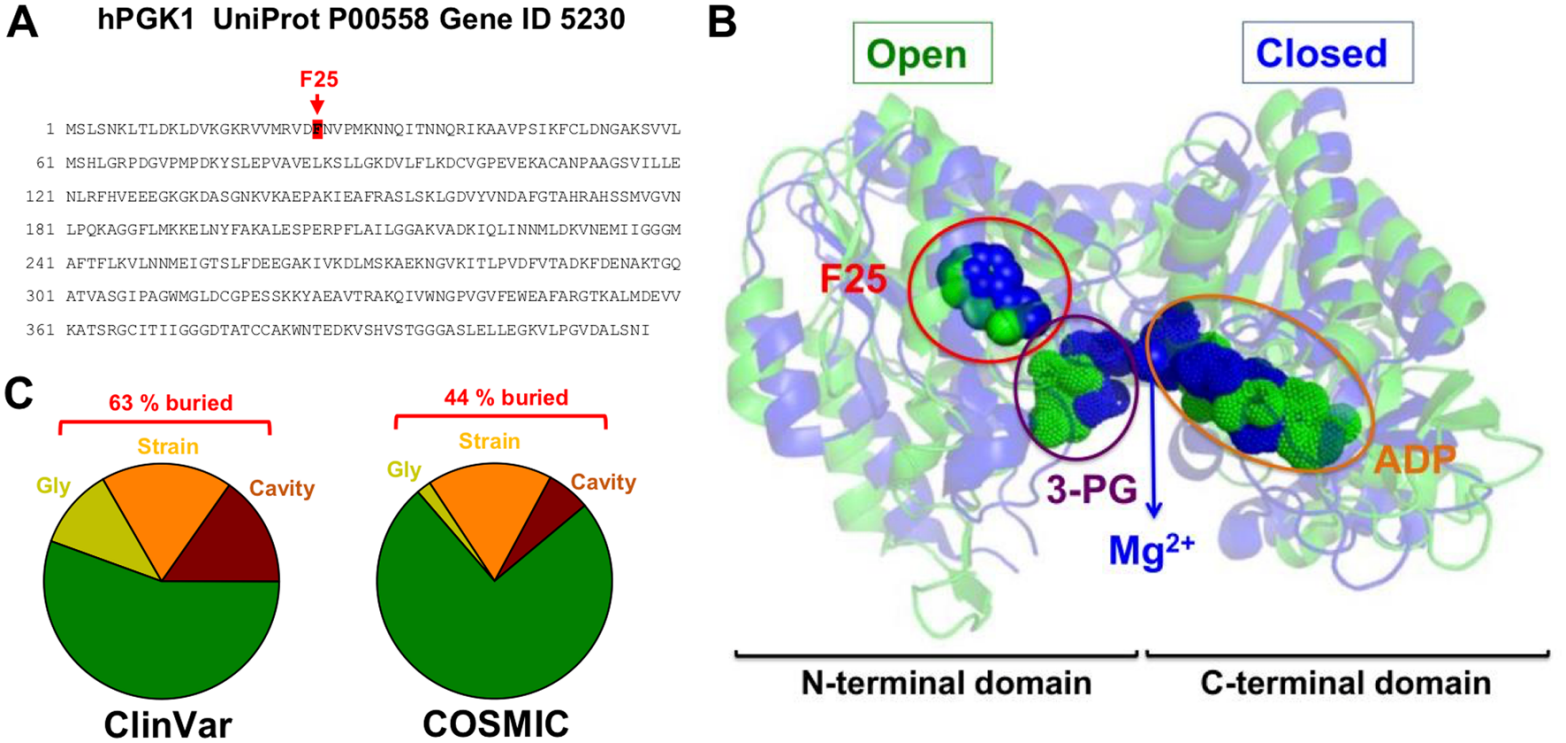
Location of F25 in the sequence and structure of hPGK1 and the biological significance of the mutations analyzed in this study. (**A**) Sequence of the hPGK1 protein (UniProt and Gene ID are indicated). The position of F25 is highlighted. (**B**) Structural location of F25 in two different conformations (open and closed) of hPGK1. The open conformation (PDB code 2XE7) is shown in green and closed conformation (PDB code 2X13) is shown in blue. These structures were reported in^14^. The location of 3-PG, ADP and Mg^2+^ is also indicated as well the two domains of the protein. Please note that the conformational transition from open to closed conformation causes changes in F25 as well as in the ligands 3-PG and ADP. (**C**) Frequency of naturally-occurring mutations in hPGK1. Data were retrieved from ClinVar and COSMIC databases, and mutations were categorized in three meaningful sets: Cavity-making (*Cavity* set, in dark red), Strain-inducing (*Strain* set, orange) or Glycine-affecting (*Glycine* set, dark yellow). The percentage of mutations belonging to either of these three sets which are buried in the structure is indicated in red. Details on the statistical classification and the identity of these mutations are found in Table S2.

Phosphoglycerate kinase (PGK) is a glycolytic enzyme that catalyzes the reversible phosphotransfer reaction between ATP and 3-phosphoglycerate (3-PG) to form ADP and 1,3 bisphosphoglycerate (1,3-BPG) in the presence of Mg^2+^^1–4^. Its main activity is to generate ATP in glycolysis, and this activity is highly conserved among all living organisms^1^. Structurally, PGK is typically a monomeric protein of about 45 kDa with two well-defined domains: an N-terminal domain (NTD) that binds 3-PG/1,3-BPG (usually the first 200 amino acids), a C-terminal domain (CTD) (typically the last 200 amino acids) that binds the nucleotide and both domains are linked by a 10–15 amino acids long α-helix (Fig. 1)^3,5^. Binding of Mg^2+^ triggers the transition between the inactive open conformation to a closed conformation that leads to catalysis^4^. Importantly, PGKs from different organisms have been crystalized in different ligation states (only for the human isoform 1, hPGK1, we retrieved 16 different structures by February 2022 from the Protein Data Bank; Table S1), and metal binding may trigger long-range cooperative communication between domains (Fig. 1). These facts make PGK a highly suitable model to investigate allosteric communication upon ligand binding (or mutations)^4,6^. Actually, due to the reversible nature of its unfolding (at least by chemical denaturants), PGK has been the subject of several key studies on folding thermodynamics, kinetics and cooperativity for a two-domain protein in vitro, inside living cells and even in multicellular organisms^7–10^. We must note that this type of studies on allosteric communication carried out at high resolution typically use small and/or single domain proteins^11–13^. Thus, this type of investigation using a two-domain and disease-associated protein can provide novel insight into the communication of mutational and ligand binding effect across the structure of a middle size and more structurally and functionally complex protein with potential biomedical implications.

Particularly in the last decade, research on hPGK1 has attracted additional attention for several reasons: (1) hPGK1 is multifunctional (a so-called *moonlighting* protein). As we have previously mentioned, it is essential for glycolysis, but it also phosphorylates other proteins^15^, it is involved in DNA replication and repair^15–17^, protein:protein interactions^15,18,19^, it activates L-nucleoside analogues used in anticancer and antiviral activities^20–23^ and plays a key role in autophagy^15^. (2) hPGK1 can operate in different cellular compartments. Obviously, due to its role in glycolysis, it is found at high levels in the cytosol. However, due to its multifunctionality, it can also localize in the nucleus^15^, mitochondria^24^ and extracellularly^25^. These findings suggest that non-native states in the energy landscape of hPGK1 may play important roles in the transport to different compartments. For instance, to be imported to mitochondria, proteins are unfolded by the mitochondrial import machinery or even presented to this machinery in a partially unfolded state by Hsp70 chaperones^26^. In the particular case of hPGK1, the interaction with Hsc70 and Hsp70 chaperones inside the cell is likely^19^, thus suggesting a role of partially (un)folded states of hPGK1 in mitochondrial import of this enzyme (this import is associated with cancer metabolism and tumorigenesis)^15^. (3) Alterations in hPGK1 activity, intracellular location and stability are associated with several diseases, including cancer^27,28^, hemolytic anemia^3^, myopathy^3^ and neurological dysfunction (possibly including amyotrophic lateral sclerosis)^3,25^. In most cases, a deficiency in hPGK1 associated with disease is concomitant with a modification of the protein sequence by mutations^2,28–31^. These mutations often reduce catalytic efficiency, protein stability and unfolding cooperativity, and increase aggregation propensity^3,28,30,31^. In several cases, a single mutation manifests this loss of function through several of these detrimental mechanisms, and mutations associated with cancer development^28^ or genetic hPGK1 deficiency (OMIM 300653)^2,3,29,30^ behave *similarly* regarding these mechanisms.

Therefore, it is plausible that missense mutations may propagate their destabilizing effect across the structure of hPGK1 affecting different *functional features* such as activity, stability, or folding cooperativity^13,32^. In this work, we analyzed the wild-type (WT) protein as well as five mutants aimed to progressively change the magnitude of the local destabilizing effect at a single site. To do so, we mutated a fully buried and large hydrophobic residue (F25; see Fig. 1 and Table S1) to the smaller residues (L, V, A and G) expected to create cavities of different size in the hydrophobic core of the NTD, increase conformational entropy (mutation to G) as well as a mutation that increases the size of the side-chain (mutation to W) expected to generate *structural strain*. These mutations are very suitable for this type of study (particularly cavity-creating mutations) because their effects on protein stability and energy landscapes have been extensively characterized by experiments and computation using typically smaller and simpler proteins^32–39^. In addition, although F25 is not found mutated in the ClinVar (https://www.ncbi.nlm.nih.gov/clinvar/) or COSMIC (https://cancer.sanger.ac.uk/cosmic) databases, a significant fraction of the mutations compiled in these databases can be classified as affecting buried positions and expected to create cavities, affecting glycine residues (i.e. altering the backbone flexibility) or introducing conformational strain (Fig. 1C and Table S2). Our approach uses a combination of experimental biophysical techniques including optical spectroscopy, calorimetry, thermal and chemical denaturation and hydrogen/deuterium exchange monitored by mass spectrometry (HDX-MS) with statistical mechanical calculations.

## Results and discussion

### F25 mutations do not impair protein expression of hPGK1 as a folded protein

**Figure 2:**
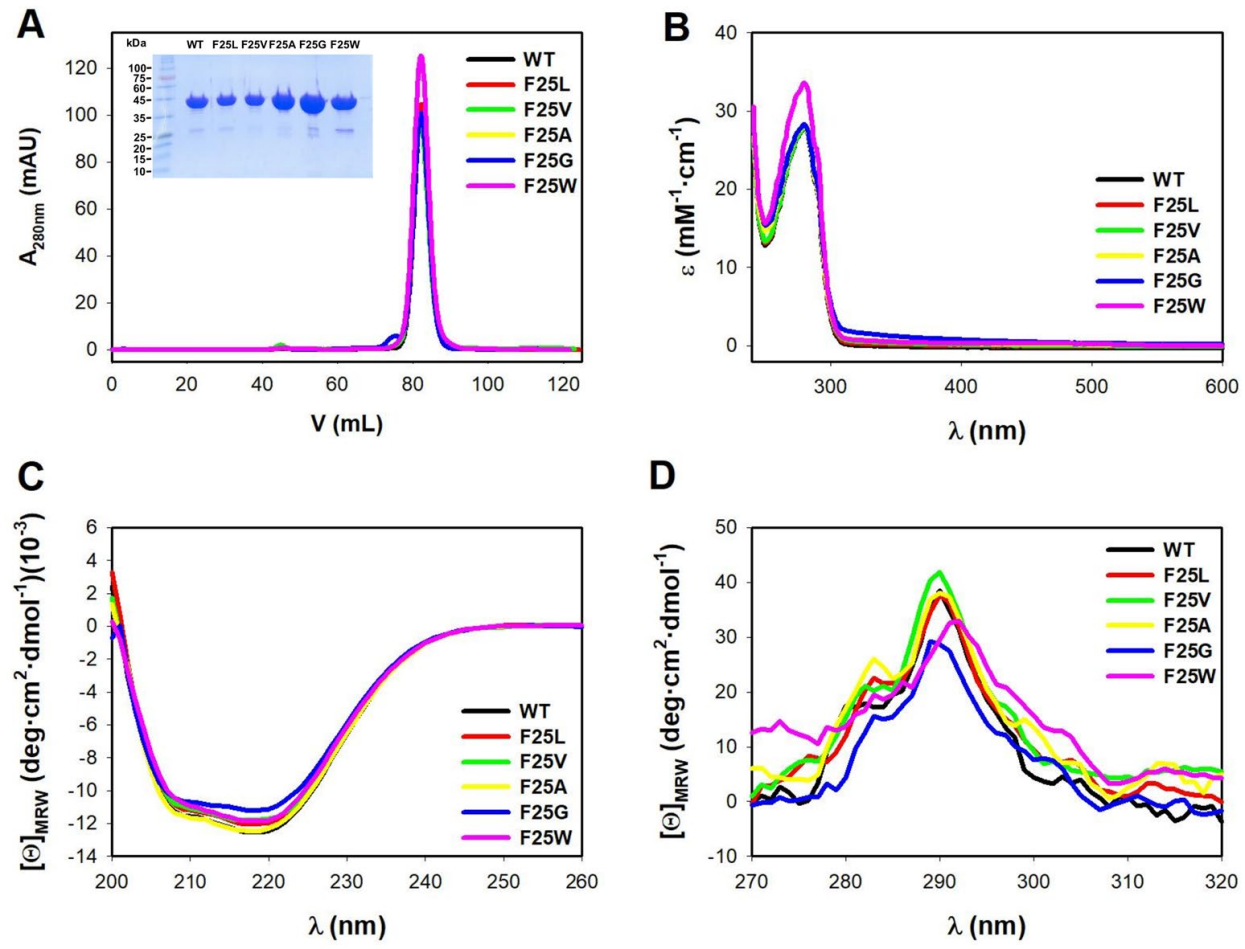
Purification and initial characterization of the conformation of hPGK1 variants. (**A**) SEC chromatograms of 4 mg of hPGK1 proteins purified from IMAC. The inset shows the corresponding SDS-PAGE (12% acrylamide) gel loaded with 5–10 µg of the protein purified from SEC. (**B**) UV–visible spectra of hPGK1 variants from absorption spectroscopy. Data were normalized considering the theoretical ε_280nm_ for each protein variant. (**C**) and (**D**) far-CD (**C**) and near-CD (**D**) spectra for each variant. Experiments in (**A**) and (**B**) were carried out in 20 mM HEPES–NaOH 200 mM NaCl, pH 7.4. In (**C**) and (**D**), experiments were performed in 20 mM K-phosphate, pH 7.4. All experiments were carried out at 25 °C. Data in (**B**)–(**D**) are the average of two independent purifications.

Upon expression in *E. coli* and purification with two chromatographic steps, IMAC (Immobilized-Metal Affinity Chromatography) and SEC (Size-Exclusion Chromatography) (Fig. 2A) we obtained hPGK1 proteins of a very high purity and also confirmed that all variants showed similar hydrodynamic behavior to that of the WT protein (i.e. monomeric; the elution volume was very similar to that of ovalbumin, with a MW of 44 kDa). Since hPGK1 is often purified with certain small molecules bound to the protein (possibly nucleotides or small polynucleotides, based on the UV–visible absorption and Circular Dichroism (CD) spectra and consistent with the ability of the enzyme to interact with such type of molecules, e.g. ADP and ATP), we therefore included a step of precipitation with streptomycin between the IMAC and the SEC. After this step, protein samples contained virtually no additional signals in the near-UV range (Fig. 2B).

Further analyses were carried out using CD to test the effect of mutations on the conformation of the protein. Far-UV CD spectra revealed the typical spectra for a protein rich in α-helix such as hPGK1 (Fig. 2C) and closely resembled results from previous studies^29,40^. We must note that the far-UV CD intensity was slightly lower for the mutant F25G, suggesting that this mutation could slightly distort the overall secondary structure of the protein. Mutants F25G and F25W also reduced the strongest signal in the near-UV CD spectra (centered at about 290 nm; consistent with the dichroic signal from Trp residues) suggesting local distortions of the tertiary structure (Fig. 2D).

These analyses support that the F25 mutants largely maintained the overall structure of the WT protein.

### Thermal stability of hPGK1 variants

**Figure 3:**
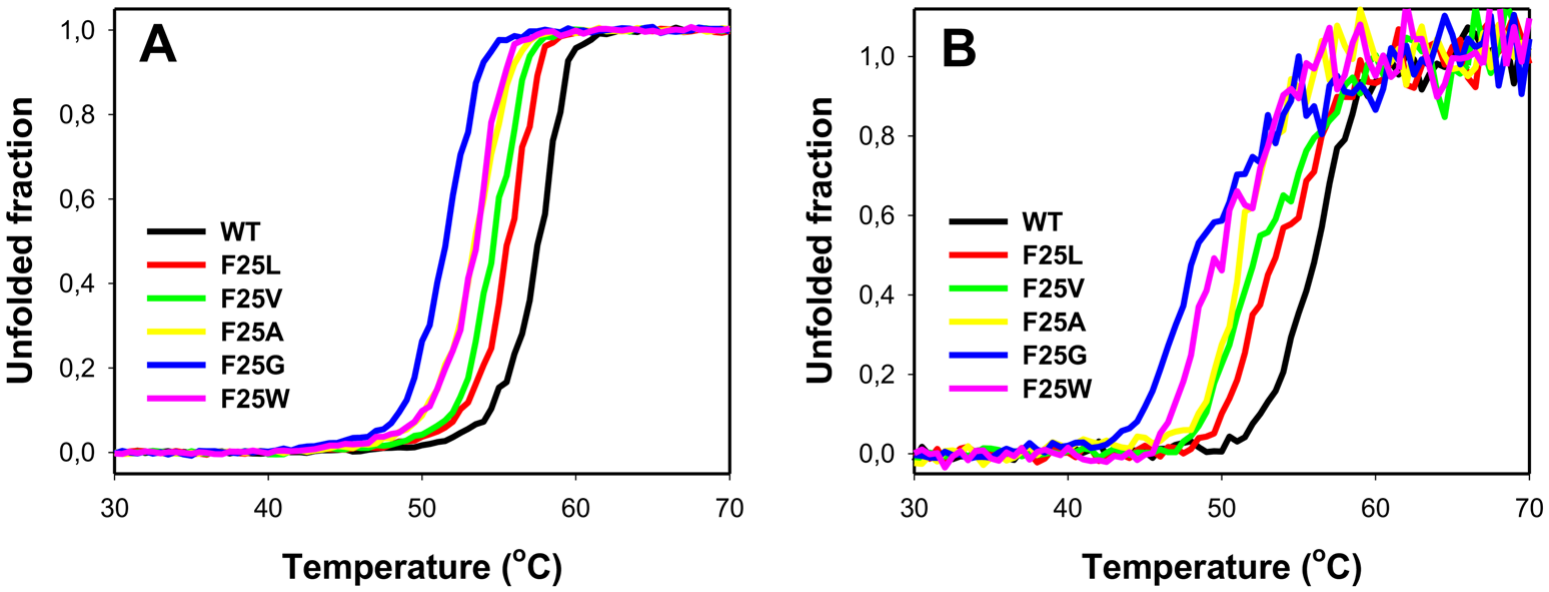
Thermal denaturation of hPGK1 variants monitored by CD spectroscopy. Signals corresponding to the Far-UV (222 nm) and the Near-UV (290 nm) are shown in panels (**A**) and (**B**), respectively. In both cases, linear pre- and post-transition baselines were applied to normalize data. Protein concentration was about 7 µM (Far-UV) and 15 µM (Near-UV). Experiments were performed in K-phosphate 20 mM, pH 7.4.

Thermal denaturation of hPGK1 has been described well by using a simple two-state irreversible denaturation model^29^. The origin of this irreversibility is likely protein aggregation, as indicated by the similar profiles obtained by DSC and light scattering measurements^30^. To initially assess the effect of mutations on hPGK1 thermal stability, we acquired the CD signal varying the temperature from 20 to 75 °C. Both far- and near-UV CD monitored thermal denaturation experiments provided similar results (Fig. 3). The apparent *T*_m_ decreased according to the size of cavity (from 2–3 °C in F25L to 6–8 °C in F25G, compared with those values of WT), whereas F25W (that may introduce certain conformational strain) showed an apparent *T*_m_ very similar to that of F25A.

**Figure 4:**
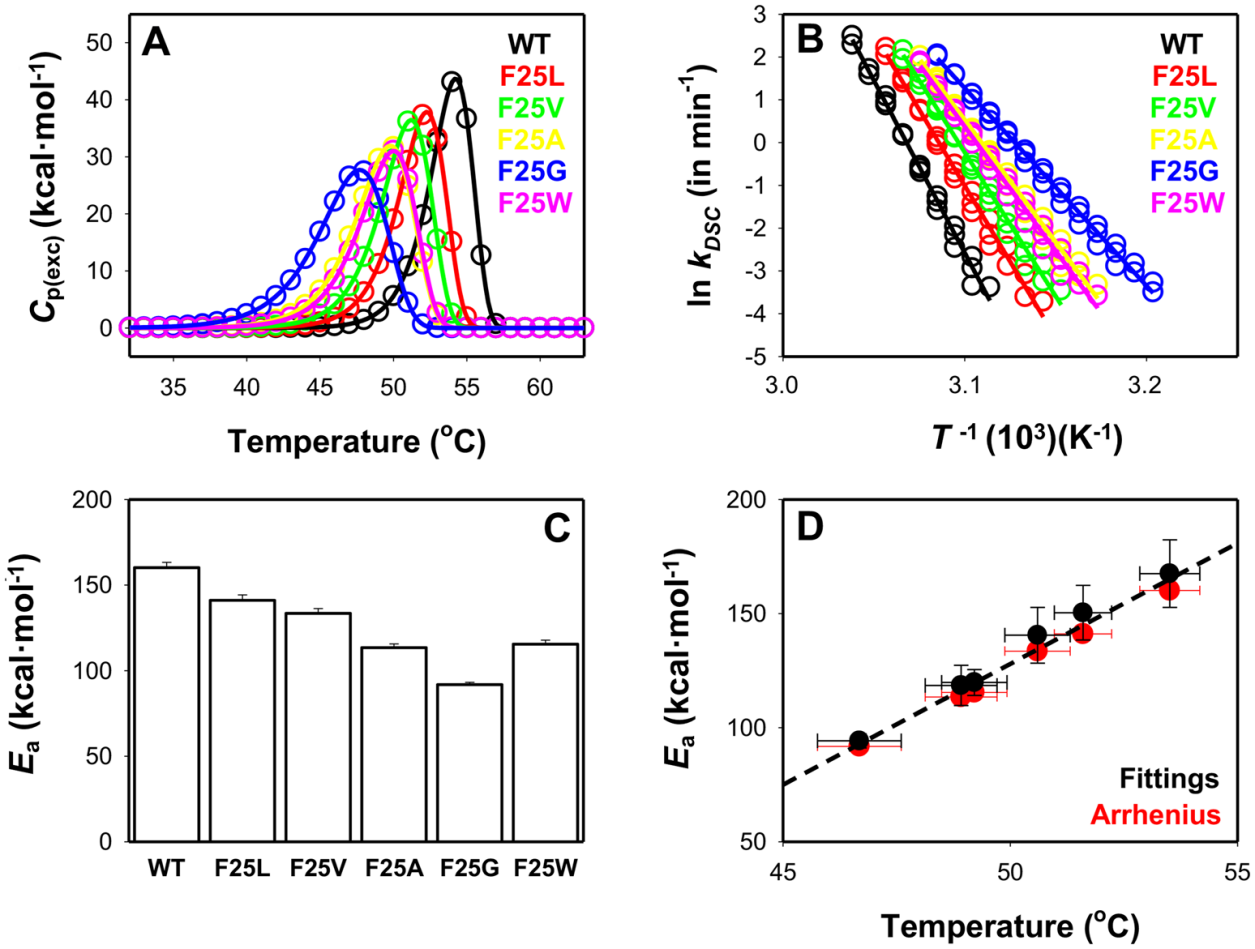
Thermal stability of hPGK1 variants by DSC. (**A**) Normalized DSC scans (upon subtraction of chemical baselines) for all variants at 10 µM protein concentration and 4 °C min^−1^. Lines are best-fits to a two-state irreversible model; (**B**) Arrhenius plot for all variants, derived from experiments at three different scan rates (1, 2.5 and 4 °C min^−1^). The *k* values were determined using Eq. (4). (**C**) Average ± s.d. for activation energies (*E*_a_ values) for the irreversible denaturation of hPGK1 variants. These values are derived from Arrhenius plots and are very consistent with those obtained from individual fittings (see Table 1). (**D**) *E*_a_ values derived from individual fittings at different scan rates (black) and from Arrhenius plots (red) are very consistent and linearly depend on the average *T*_m_ from individual fittings (x-axis). Errors for *fittings* are S.D. for different experiments whereas those for *Arrhenius* are those from linear fittings (see panel **B**). Experiments were carried out in 20 mM HEPES–NaOH 200 mM NaCl, pH 7.4. All the analysis were carried out following^29,41^.

To provide deeper knowledge of the effects of F25 mutants on the kinetic stability (i.e. the rate constant for irreversible denaturation, *k* and activation energy, *E*_a_) of hPGK1, we applied DSC using previously described protocols for this protein^31^. The results from these analyses are summarized in Fig. 4 and Tables 1 and 2. In agreement with thermal denaturation monitored by CD, we observed a progressive decrease in *T*_m_ in the cavity-making mutants (F25G decreases the *T*_m_ by ~ 7 °C). The mutant F25W showed again a similar behavior to that of F25A, with a decrease of ~ 4.5 °C. The destabilization induced in the thermal denaturation process by each individual mutation using different probes (DSC, far-UV and near-UV CD spectroscopies) are very close at similar scan rates (Figs. 3 and S1–S2).

**Table 1:** Kinetic parameters derived from Arrhenius plots calculated from DSC data in the absence of urea.

Variant	Parameter			
	*E*_a_ (kcal·mol^−1^)	Δ*E*_a_ (kcal·mol^−1^)	*k*_37 °C_ (min^−1^)	ΔΔ*G*^≠^_37 °C (var-WT)_(kcal·mol^−1^)
WT	168.0 ± 3.3	0.0 ± 4.7	1.8 ± 0.4·10^−6^	0.00 ± 0.19
F25L	149.3 ± 3.1	− 18.7 ± 4.4	2.9 ± 0.6·10^−5^	− 1.71 ± 0.19
F25V	136.9 ± 2.7	− 31.1 ± 3.8	1.5 ± 0.2·10^−4^	− 2.73 ± 0.16
F25A	114.3 ± 2.1	− 53.7 ± 3.0	1.3 ± 0.1·10^−3^	− 4.06 ± 0.14
F25G	92.9 ± 1.2	− 75.1 ± 3.5	1.1 ± 0.1·10^−2^	− 5.37 ± 0.15
F25W	117.8 ± 2.1	− 50.2 ± 3.0	9.2 ± 1.0·10^−4^	− 3.84 ± 0.15

*k*_37 °C_ was obtained using 37 °C (310.15 K) as reference temperature (T*) in Eq. (2).

**Table 2:** Energetic parameters for the denaturation of hPGK1 variants determined from DSC data in the absence or the presence of urea.

Variant	Urea (M)	*T*_m_ (°C )	Δ*H* (kcal·mol^−1^)	*E*_a_ (kcal·mol^−1^)
WT	0	53.5 ± 0.7	162 ± 10	168 ± 15
	0.5	51.2 ± 0.7	142 ± 12	140 ± 7
	1	48.7 ± 0.9	136 ± 13	115 ± 4
F25L	0	51.6 ± 0.6	156 ± 6	150 ± 12
	0.5	49.2 ± 0.7	133 ± 6	129 ± 4
	1	46.4 ± 0.9	126 ± 16	105 ± 1
F25V	0	50.4 ± 0.7	153 ± 11	140 ± 12
	0.5	47.5 ± 0.9	133 ± 14	106 ± 5
	1.1	44.3 ± 1.3	114 ± 22	80 ± 2
F25A	0	48.9 ± 0.8	153 ± 14	119 ± 9
	0.5	45.8 ± 1.1	124 ± 16	89 ± 3
	1	42.8 ± 1.7	109 ± 18	68 ± 3
F25G	0	46.7 ± 0.9	155 ± 15	94 ± 3
	0.5	43.5 ± 1.6	123 ± 11	71 ± 4
	1	41.1 ± 1.9	108 ± 19	59 ± 5
F25W	0	49.2 ± 0.7	140 ± 17	120 ± 6
	0.5	46.3 ± 0.9	120 ± 18	93 ± 2
	1	43.0 ± 1.5	96 ± 16	72 ± 5

Data are the mean ± s.d. for three different scan rates (4, 2.5 and 1 °C min^−1^) at 7–15 µM protein concentration and using a two-state irreversible model.

It is also interesting to note that the decrease in thermal stability correlates strongly with the *E*_a_ values for irreversible denaturation and the *T*_m_ values as determined by DSC (Fig. 4B–D and Tables 1 and 2). An overall linear fitting of *E*_a_ versus *T*_m_ provides a value for the change in activation heat capacity (Δ*C*_p_^≠^) of 10.6 ± 0.5 kcal mol^−1^ in a very good agreement with our previous analyses that yielded a value of 9.1 ± 0.8 kcal mol^−1^^29^ (Fig. 4D). Notably, the mutation F25G decreased the *E*_a_ value by 75 kcal mol^−1^ (basically to a half of that for the WT protein).

These results have remarkable implications for the kinetic stability of hPGK1 variants in vitro, since the decrease in *T*_m_ values correlated with the decrease in *E*_a_ values, and thus, the extrapolated values of the kinetic rate constants at 37 °C (and therefore, the associated half-lives for irreversible denaturation) vary strongly among variants. We observed a gradual decrease of the half-life for irreversible denaturation correlating with the size of the cavity, from a small ~ 15-fold in F25L to a large ~ 6000-fold in F25G. Again, F25A and F25W showed similarly low kinetic stabilities (about 600-fold lower than that of the WT protein; extrapolated to 37 °C from Arrhenius plots) (Table 1).

It is worth noting that previous studies have shown that mutations associated with hPGK1 deficiency (a rare X-linked genetic disorder^3,30^) also showed different effects on kinetic stability when extrapolated to 37 °C, up to 5 orders of magnitude lower than that of the WT protein in some cases. Interestingly, mutations found in COSMIC database also showed reduced stability against thermal and chemical denaturation^28^.

To further investigate the correlation found between the changes in *T*_m_ and *E*_a_ values, we have carried out DSC experiments in the presence of urea (Table 2 and Figs. S1–S2). These analyses allow to extract the kinetic *m*^≠^ value for irreversible denaturation from detailed DSC analyses at different scan rates and urea concentrations^29,31,41^. Comparing this value with the theoretical *m* value derived from the protein size (i.e. in number of amino acids), we can estimate the degree of the native hPGK1 structure which is denatured in the transition state for irreversible thermal denaturation (Table 3). Actually, these analyses revealed that, in contrast to hPGK1-deficiency causing mutants characterized so far^29^, only the F25G showed a small decrease in value for the *m*^≠^ (Table 3). Thus, the Hammond effect (i.e. that the kinetic stability decreases as the transition state becomes more native-like) proposed for the hPGK1-deficiency causing mutants previously characterized^29^ does not seem to follow the same behavior that those of the F25 mutants characterized in the present work (Table 3).

**Table 3:** Degree of structural unfolding in the transition state for the irreversible denaturation of hPGK1 variants determined by DSC.

Variant	*m*^≠^ (kcal·mol^−1^·M^−1^)	*m*^≠^/*m*_unf_
WT	2.69 ± 0.36	0.59 ± 0.08
F25L	2.47 ± 0.34	0.54 ± 0.07
F25V	2.74 ± 0.51	0.60 ± 0.11
F25A	2.60 ± 0.60	0.57 ± 0.13
F25G	1.93 ± 0.42	0.43 ± 0.09
F25W	2.63 ± 0.48	0.58 ± 0.11

*m*^≠^ values were determined from DSC experiments and Eq. (5). *m*_unf_ is the theoretical value (4.54 kcal mol^−1^ M^−1^) for the full unfolding of the protein^29^ based on a protein size of 417 residues^42^.

### Urea denaturation of F25 mutants reveals effects on both unfolding cooperativity and resistance to the urea-induced unfolding

An interesting feature of PGKs is that their chemical denaturation is generally reversible (e.g. using urea or guanidium chloride)^8,29,43^. Therefore, this type of analysis is amenable for comparing the effects of mutations on thermodynamic stability and unfolding cooperativity. Urea-denaturation of WT hPGK1 resembles quite well a two-state folder^28,29^ although the equilibrium *m* value is somewhat lower than the expected for a protein of this size. Importantly, urea-induced denaturation of 14 mutants, including those found in cancer cell lines and hPGK1-deficient patients, shows reduced unfolding cooperativity (in some cases the *m*_urea_ value was half of that of the WT protein). Interestingly, only in a few cases changes in the *C*_m_ values were observed^28–30^.

**Figure 5:**
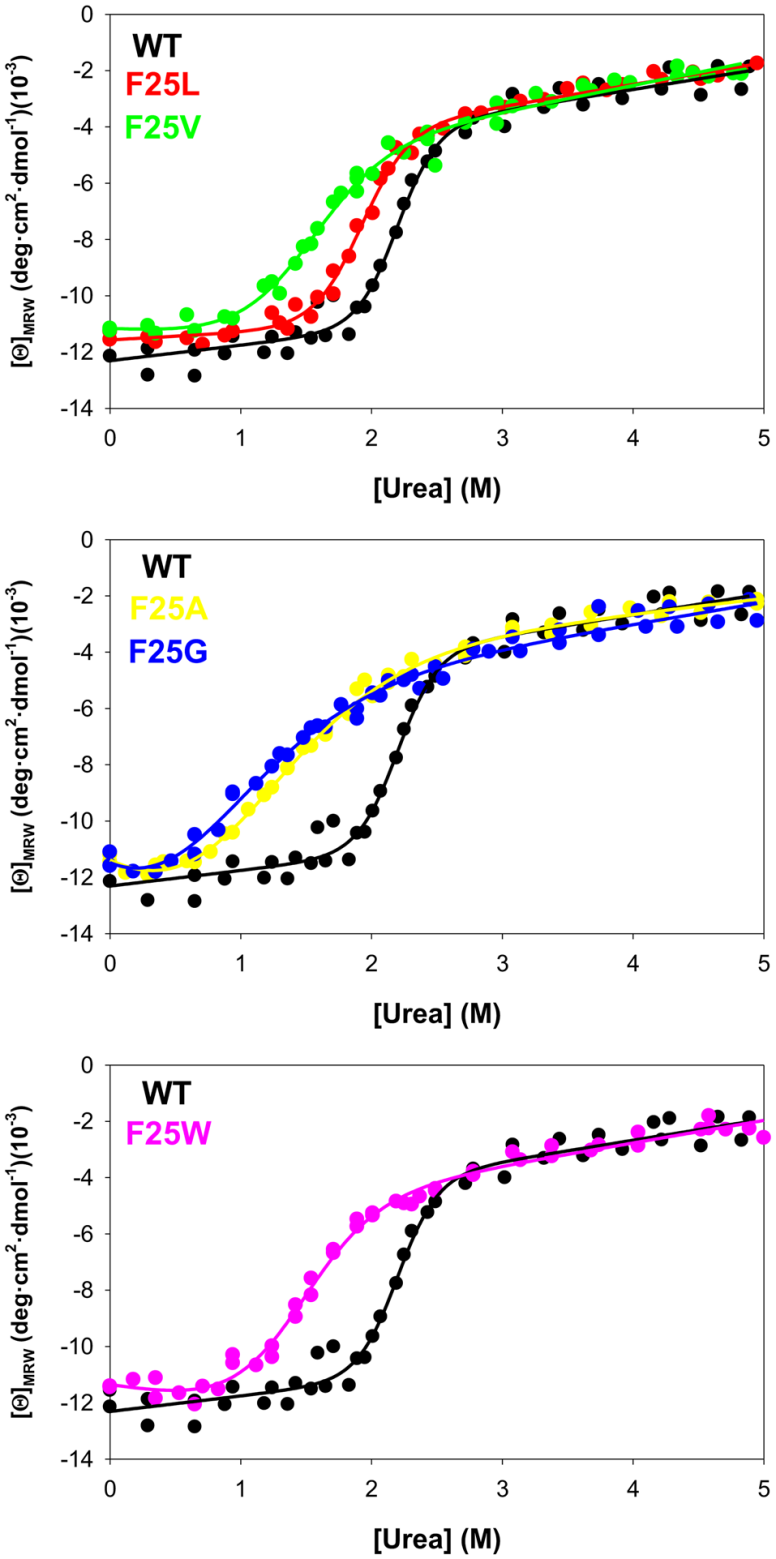
Urea-induced denaturation of hPGK1 variants at 25 °C. 5 µM of hPGK1 variants were incubated for at least 2 h in the presence of increasing urea concentrations in HEPES–NaOH 20 mM NaCl 200 mM 1 mM β-ME, pH 7.4. Then the far-UV CD spectra were acquired and appropriate blanks subtracted. Data are from two independent experiments, and in each experiment the sample without urea was triplicated and averaged. Lines are best-fits to an apparent two-state equilibrium denaturation model (Eq. 6).

To compare the behavior of F25 mutants versus those of naturally-occurring and disease-associated, we carried out similar urea-induced unfolding experiments (Fig. 5). As previously described, denaturation of hPGK1 provides a value for the unfolding free energy of about 8 kcal mol^−1^, based on the linear extrapolation method (Table 4). The F25 mutants showed changes in urea-induced unfolding compared to the WT protein. The mutants F25L and F25V, as well as the strain-inducing F25W, caused a significant decrease in the unfolding cooperativity and *C*_m_ values (note that in this case F25W resembles F25V). Some mutants decreased the *m*_urea_ value by ≤ 50% of the value for the WT protein (Table 4). The most destabilizing variants were F25A (for which we could obtain reasonable and physically meaningful values) and F25G (that showed a denaturation profile similar to that of F25A but we could not get reliable estimations of *C*_m_ or *m*_urea_, see Table 4). Therefore, for F25G and F25A, the unfolding cooperativity was severely reduced. Overall, these results suggest that increasing the cavity size or introducing conformational strain, at least at the F25 site in hPGK1, increase the population of non-native state conformations, possibly even under native conditions. We further tested this hypothesis by carrying out additional experimental and computational analyses under native conditions.

**Table 4:** Apparent denaturation equilibrium parameters with urea at 25 °C determined by Far-UV CD.

	WT	F25L	F25V	F25A	F25G	F25W
*m*_urea_ (kcal·mol^−1^·M^−1^)	3.86 ± 0.63	3.24 ± 0.30	2.05 ± 0.31	1.21 ± 0.12	≤ 1	2.07 ± 0.26
*C*_m_ (M)	2.20 ± 0.03	1.92 ± 0.02	1.50 ± 0.08	0.89 ± 0.17	~ 1	1.42 ± 0.06

*C*_m_ and *m*_urea_ values were retrieved from fittings of experiments shown in Fig. 5. For F25G, we could only obtained reasonable fittings when *C*_m_ or *m*_urea_ values were fixed, and thus some gross estimation was only possible.

### Proteolysis suggests a mild increase in the population of partially folded states in the hPGK1 mutants under native conditions

**Figure 6:**
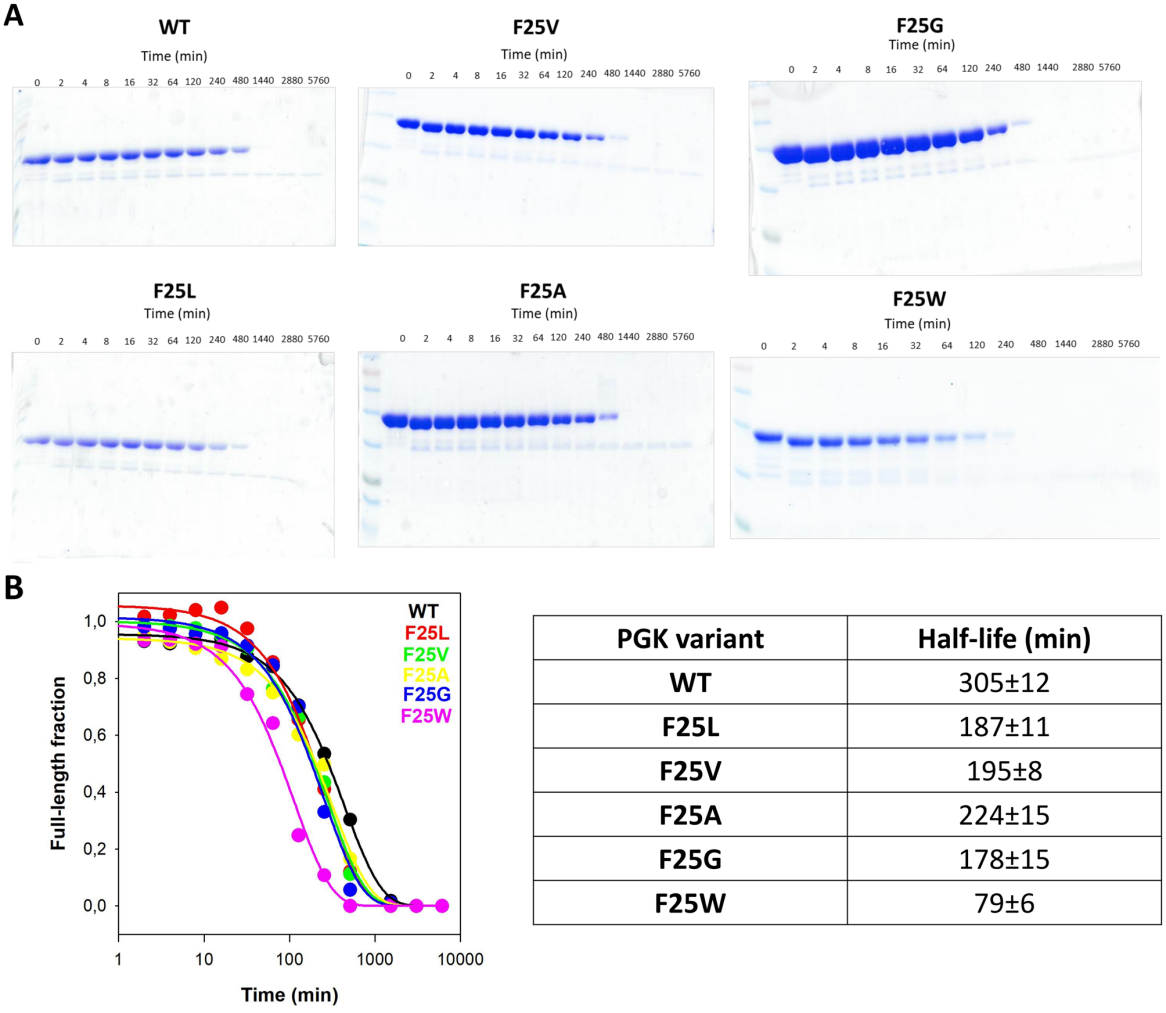
Conformational stability of WT and F25 mutants as determined by proteolysis with thermolysin. (**A**) Representative SDS-PAGE gels of hPGK1 variants proteolysis kinetics; (**B**) Densitometric analysis of results shown in panel A with fittings to a single exponential function (left panel) and the corresponding half-lives (right panel). Thermolysin concentration was 1 µM. Experiments were carried out in 20 mM HEPES–NaOH 200 mM NaCl, pH 7.4 at 25 °C.

Proteolysis has been shown to provide information on the population of partially (un)folded, proteolytic sensitive substates in the native state ensemble of hPGK1^29,30^. At low protease concentrations, the overall proteolytic rate for the WT protein is dependent on the protease concentration (Figs. 6 and S4)^31^ and thus, effects on this rate constant reflect to some extent the increased population of partially unfolded (i.e. cleavable) substates under native conditions. Interestingly, mutants F25L, F25V and F25A showed modest effects on proteolysis (1.3–1.8-fold increased sensitivity; Figs. 6 and S4) while mutations F25G and F25W showed more clear effects (1.7–2.6-fold and 3.9–5.3-fold increase, respectively). Interestingly, the latter mutants showed overall proteolytic rates rather insensitive to protease concentration (Figs. 6 and S4) suggesting that in these two variants, the unfolding rate constant to the cleavable state may dominate the overall proteolytic rate constant.

### Perturbations in the native ensemble of F25 mutants monitored by HDX-MS

**Figure 7:**
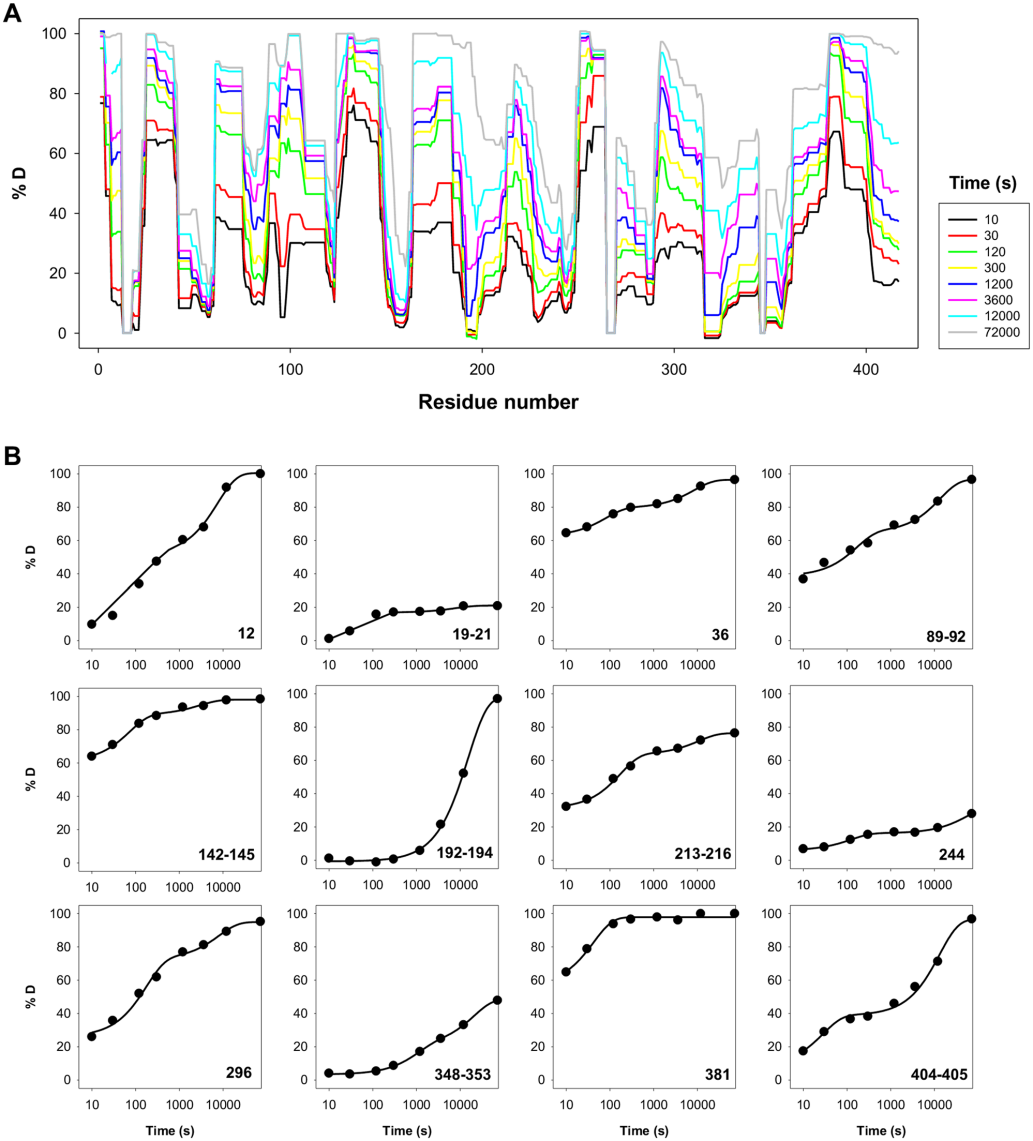
Kinetics of deuterium (D) incorporation to hPGK1 WT. (**A**) % of D incorporation at different time points for the entire hPGK1 protein. Note that segments 13–17, 265–269 and 345–347 were not detected. (**B**) Selected segments displaying complex kinetics. Lines generally show best-fits to a double-exponential function including a burst phases. The best-fit parameters are compiled in Table 5. Experiments were carried out in 20 mM HEPES–NaOH, 200 mM NaCl, 0.5 mM TCEP, pD 7.4 at 25 °C.

To provide experimental information on the effects of F25 mutations on the local stability of the native state ensemble, we carried out HDX-MS experiments. Analysis of the WT protein in a ligand-free conformation (Fig. 7) provided some interesting results. First, the apparent local stability based on the overall exchange rate for diverse protein segments is highly different (Fig. 7A). Second, the exchange kinetics are complex, and phenomenologically described in many cases by a double exponential function with a burst-phase (Fig. 7B and Table 5). This suggests that different conformational substates are populated in the native state ensemble of hPGK1.

**Table 5:** Best-fit parameters for selected segments of WT hPGK1 determined by HDXMS.

Parameter	*A*_∞_ (%D)	*A*_1_ (%D)	*k*_1_ (s^−1^)	*A*_2_ (%D)	*k*_2_ (s^−1^)	*r*^2^
Segment						
12	100.5 ± 1.8	45.1 ± 2.7	7.2 ± 1.1·10^−3^	48.9 ± 2.7	1.3 ± 0.2·10^−4^	0.9986
19–21	21.1 ± 0.7	19.6 ± 1.2	2.0 ± 0.4·10^−2^	4.2 ± 0.9	1.2 ± 0.7·10^−4^	0.9956
36	96.5 ± 0.3	16.9 ± 0.4	1.3 ± 0.1·10^−2^	17.0 ± 0.3	1.2 ± 0.1·10^−4^	0.9998
89–92	96.6 ± 3.7	26.4 ± 4.7	6.1 ± 2.9·10^−3^	31.7 ± 5.0	7.5 ± 3.2·10^−5^	0.9858
142–145	98.0 ± 1.0	28.1 ± 2.0	1.4 ± 0.3·10^−2^	9.2 ± 1.9	3.4 ± 1.7·10^−4^	0.9957
192–194*	97.8 ± 1.4	98.5 ± 1.5	6.5 ± 0.3·10^−5^	N.app	N.app	0.9990
213–216	76.4 ± 1.4	32.4 ± 1.9	5.8 ± 0.9·10^−3^	12.9 ± 2.0	9.3 ± 3.8·10^−5^	0.9968
244	32.2 ± 3.1	10.3 ± 0.5	8.1 ± 1.1·10^−3^	16.0 ± 3.0	1.8 ± 0.5·10^−5^	0.9986
296	94.9 ± 3.2	46.6 ± 4.7	5.7 ± 1.4·10^−3^	22.3 ± 4.8	1.2 ± 0.7·10^−4^	0.9931
348–353	48.7 ± 0.6	17.4 ± 1.1	1.0 ± 0.1·10^−3^	27.9 ± 1.0	4.8 ± 0.6·10^−5^	0.9997
381*	97.8 ± 0.8	41.1 ± 3.6	2.4 ± 0.5·10^−2^	N.app	N.app	0.9836
404–405	96.5 ± 3.3	30.2 ± 8.0	3.6 ± 1.8·10^−2^	57.8 ± 3.7	7.7 ± 1.4·10^−5^	0.9934

Good fittings were obtained using a two-exponential function (*A*_∞_ is the total amplitude of %D uptaken at *t* = ∞, *A*_1_ and *k*_1_ are the amplitude and rate constant for the fast phase, while *A*_2_ and *k*_2_ are those for the slow phase).

*The fitting to a double-exponential function did not converge. The values reported corresponded to a fitting using a one-exponential function with a burst-phase. N. app.- not applicable.

**Figure 8:**
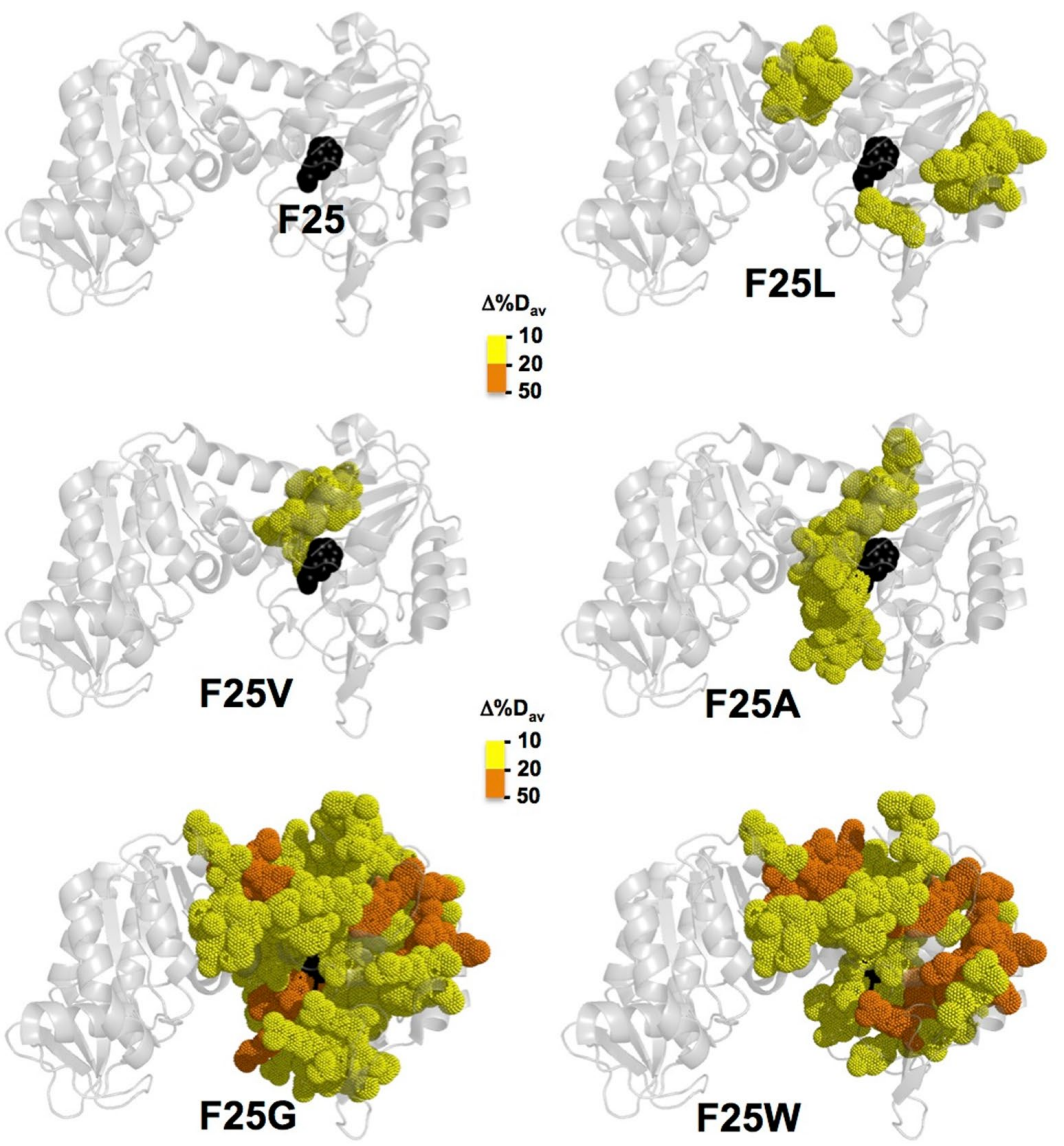
Structural destabilization of hPGK1 due to F25 mutations as determined by HDX-MS. The value of Δ%D_av_ was calculated using the WT protein as reference and represented as shown in the color scale. The structure used for visualization corresponds to the open conformation (PDB code: 2XE7)^14^.

Similar experiments were carried out with the F25 mutants. F25 mutations selectively affected the stability of certain regions in the protein (Figs. S5–S9). Mutants F25L and F25V showed mild stability decreases in regions close to F25, while these effects were more extensive due to the mutation F25A (Fig. 8). Mutants F25G and F25W showed the strongest effects in stability and these propagated to regions further from the mutated sites, particularly relevant in the domain-domain interface (Figs. 8 and S10, 11).

The kinetic stability (as determined by DSC; Tables 1, 2 and 3) and unfolding cooperativity (as determined by isothermal urea denaturation; Table 4) are likely associated with the interaction surface between the N- and C-terminal domains (NTD and CTD, respectively) (see Figs. S10 and S11). We next analyzed the effects of the F25 mutations on this interface from HDX-MS data (Fig. S11). These analyses showed that even the mutation F25V begins to perturb this interface, and mutants F25G and F25W have the largest effect, affecting 30–40% of the residues forming this interface (Fig. S11A) and these effects are of larger magnitude (Fig. S11B). These results strongly suggest that the effects of F25 mutations on conformational stability and unfolding cooperativity can be, at least, partially explained by the structural destabilization of the NTD: CTD interface.

### Statistical mechanical analysis of the effects of F25 mutations

**Figure 9:**
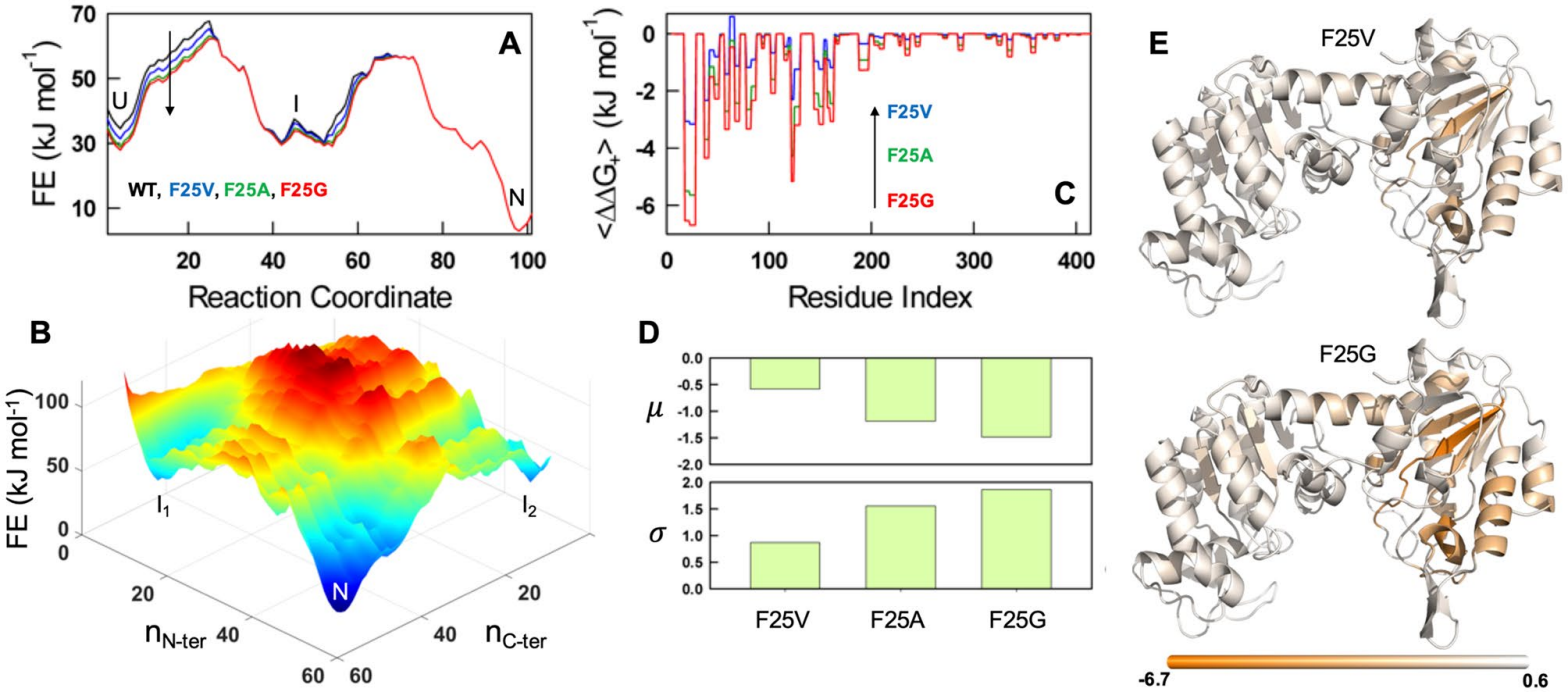
Statistical mechanical predictions for WT and F25 mutants using a WSME model. (**A**) One-dimensional free energy (FE) profiles of the selected variants as a function of number of structured blocks (the reaction coordinate). U, I and N stand for unfolded, intermediate and native states, respectively. The arrow indicates the direction of destabilization with F25G (red) being the most destabilized. (**B**) Two-dimensional conformational landscape highlighting the presence of two intermediates, I_1_ and I_2_, when plotted as a function of the number of structured blocks in the NTD (n_N-ter_) and the CTD (n_C-ter_). (**C**) Perturbation profiles of the F25 variants with F25G being the most perturbed mutant and showing that the perturbation is localized to the NTD. (**D**) Mean and standard deviation of the perturbation profile shown in panel (**C**). (**E**) Cartoons showing perturbations from panel (**C**) mapped onto the structure. It can be seen that the NTD is more perturbed in the F25G variant (darker orange over a larger fraction of residues) when compared to the F25V mutant. The color scale in panel (**E**) corresponds to the values for < ΔΔG_+_ > in kJ·mol^−1^ (see panel (** C**)).

To further understand the extent to which mutations affect the different regions of the protein, we introduced mutations in silico (using PyMol) and studied three representative F25 mutants (F25V, F25A and F25G) via the WSME model. In each of the cases, no further modulation of structure was introduced as these are primarily truncating mutations. The mutated structures were fed into the WSME model and the free-energy profiles were predicted without changing any model parameters. The resulting ranking of destabilization matched the expectations from experiments, i.e. F25G > F25A > F25V (Fig. 9A) and also indicated that the folded state of the NTD and CTD is uncoupled to some extent in the native state ensemble of hPGK1 (intermediates I_1_ and I_2_, Fig. 9B). In addition, we calculated the positive coupling free energies ($\Delta {G}_{+}$) for these variants, a parameter that reports on the degree of relative thermodynamic coupling between residues that are folded^44^. The effect of each of the mutations (*mut*) versus the WT protein results in the mean difference in coupling free energies $\langle {\Delta \Delta G}_{+}\rangle$ or perturbation, defined as $\Delta {G}_{+,mut}-\Delta {G}_{+,WT}$, (plotted as a function of the residue number; Fig. 9C). These results show a similar ranking of the mutations (F25G > F25A > F25V) according to their negative impact on thermodynamic coupling of folded regions in the native state ensemble.

As expected from the decoupled nature of the two domains, the perturbations are mostly localized to the NTD mirroring experimental observations. The mean perturbation magnitude ($\mu$), i.e. the average of the values in Fig. 9C corresponding to the first 200 residues (N-terminal domain), follows experimental HDX patterns with the F25G being the most perturbed (Fig. 9D, top panel). The corresponding standard deviation in perturbation ($\sigma$), that measures the extent of distribution, followed a similar trend (Fig. 9D, bottom panel), highlighting that the origins of the complex HDX-MS kinetics are a manifestation of population redistribution in the conformational landscape. The perturbation, when mapped onto the structure, vividly reveals the extent to which a single mutation affects neighbouring residues and the extent of coupling between them (Fig. 9E).

It is interesting to note that local stability measurements using HDX-MS (Fig. 8) and statistical mechanical model (Fig. 9) show that destabilization caused by F25 mutations are mostly restricted to the NTD and increases with the predicted size of the cavity (F → L < F → V < F → A) and conformational entropy (F → A < F → G). These analyses indicate that gradual local destabilization (due to alterations of the interaction networks) is connected to the effects on the global stability (i.e. reversible by urea or kinetically-controlled by temperature) and unfolding cooperativity (Figs. 4 and 5 and Tables 1, 2 and 4). Since hPGK1 WT resembles a two-state (un)-folder by urea, our results support that it displays certain heterogeneity of substates populated in the native state ensemble. It is likely that F25 mutations enhance such heterogeneity shifting the equilibrium towards partially unfolded substates, thus accelerating HDX and reducing the apparent unfolding cooperativity. This link between local destabilization of the NTD and global denaturation effects (i.e. thermal and chemical denaturation) could be also partially explained by the effects of the F25 mutations on the NTD: CTD interface (Fig. S11).

## Conclusions

Understanding how mutations affect the native state ensemble is essential to obtain insight into mutational effects on protein stability and function as well as their potential pathological consequences^45,46^. This is a challenging task since proteins display multiple features (stability, enzymatic function, regulation, biomacromolecular interactions, different subcellular locations, etc.) and a large genetic diversity exists in human population^45–47^. In this work, we have investigated how mutations targeting a fully buried Phe residue propagate their effects across the structure of the model two-domain hPGK1 enzyme, associated with disease^3,28,30^. In our strategy, we progressively decreased the size of the side-chain (F → L, F → V, F → A), additionally increased the entropy of the side chain (F → G) or potentially introduced conformational strain (F → W). A combination of biophysical studies (thermal and chemical denaturation, proteolysis), HDX-MS and statistical mechanical calculations showed that even small local perturbations (e.g. F → L or F → V) have remarkable effects on the unfolding cooperativity and energetics. Intriguingly, the mutation aimed to introduce conformational strain (F → W) is as damaging to conformational stability of hPGK1 as the mutation F → A and slightly less than the mutation F → G, with severe effects on thermal/kinetic stability, denaturation energetics, unfolding cooperativity and local stability of the NTD. Although mutations creating a protein cavity or introducing conformational strain could show similar effects on protein stability^32^, in our case a mutation that would increase by ~ 20% the size of the side-chain (F25W) shows comparable effects that one reducing this size by ~ 50% (F25A)(both based on the average volumes reported by^48^). The molecular origin of this apparently stronger effect of conformational strain is unclear.

Although this set of F25 mutations has not direct relationship with disease (i.e. not found in ClinVar or COSMIC databases), it represents a large fraction of naturally-occurring missense variants (Fig. 1C and Table S2). It is also worth noting that COSMIC mutations in hPGK1 (e.g. V216F and F241S)^28^ as well as several mutations analyzed from inherited hPGK1 deficiency leading to changes in the size-chain or the conformational entropy of the protein backbone (e.g. L89P and S320N)^30^ show effects on hPGK1 conformational landscape consistent with those reported here for the F25 mutants. We propose that our approach, combining biophysical analyses, HDX-MS studies and statistical mechanical calculations may allow to obtain high-resolution conformational information on the effects of cancer-associated mutations^28^, mutations associated with hPGK1 deficiency^3,30^ and cancer-associated post-translational modifications^49^. In addition, alterations in protein dynamics (in the ns-ms time scale) might be evaluated by molecular dynamics simulations^50–52^. We expect that similar analyses on mutations found in COSMIC (https://cancer.sanger.ac.uk/cosmic/gene/analysis?ln=PGK1_ENST00000644362), OMIM (https://omim.org/entry/311800) and gnomAD (https://gnomad.broadinstitute.org/gene/ENSG00000102144) databases will allow us to generate predictive tools to discern between pathogenic and neutral mutations^47^.

## Material and methods

### Protein expression and purification

hPGK1 WT cDNA was chemically synthesized by GenScript (Leiden, The Netherlands) and cloned into the pET15b plasmid. The cloning strategy introduced in the N-terminal Met1 of the hPGK1 WT the following sequence: MGSSHHHHHHSSG**LVPRGS**H. Residues underlined constitute the His_6_-tag used to purify the proteins by immobilized-metal affinity chromatography (IMAC) and the sequence in bold is the thrombin cleavage site. Mutants were generated by standard site-directed mutagenesis (GenScript, Leiden, The Netherlands). pET15b-hPGK1 plasmids were used to transform BL21(DE3) *E. coli* cells (Agilent Technologies, Santa Clara, CA, USA) for protein expression. For each variant, 40 mL of Luria–Bertani (LB) medium containing 0.1 mg mL^−1^ ampicillin (Canvax Biotech, Córdoba, Spain) were inoculated with transformed cells and grown for 16 h at 37 °C. These cultures were diluted into 800 mL of LB containing 0.1 mg mL^−1^ ampicillin, grown at 37 °C for 3 h to reach an optical density of about 1.0 and then these were transferred to 25 °C and induced with 0.5 mM IPTG (Isopropyl β-d-1-thiogalactopyranoside, Canvax Biotech, Córdoba, Spain). After 6 h, bacteria were harvested by centrifugation and frozen at − 80 °C overnight. Cells were resuspended in binding buffer, BB (20 mM Na-phosphate, 300 mM NaCl, 50 mM imidazole, pH 7.4) containing 1 mM PMSF (phenylmethylsulfonyl fluoride, Sigma-Aldrich) and sonicated in an ice bath. These extracts were centrifuged (20,000 g, 30 min, 4 °C) and the supernatants were loaded into IMAC (Ni-Sepharose immobilized-metal affinity chromatography, from Cytiva, Barcelona, Spain) columns, washed with 40 bed volumes of BB and eluted in BB containing a final imidazole concentration of 500 mM. These eluates were treated with streptomycin 10% W/V to remove (poly)-nucleotides that may be bound to the hPGK1 protein. After 30 min of centrifugation at 20,000 g and 4 °C, supernatants were loaded into PD-10 columns (Cytiva, Barcelona, Spain) and buffer exchanged to the storage buffer (20 mM HEPES–NaOH, 200 mM NaCl, pH 7.4). These samples were flash-frozen in liquid N_2_ and stored at − 80 °C.

To remove protein aggregates and increase protein purity, proteins were subjected to size exclusion chromatography on a Superdex 200 16/60 column (GE Healthcare) using 20 mM HEPES–NaOH, 200 mM NaCl, pH 7.4 as mobile phase at 1.5 mL min^−1^ flow rate. Monomeric fractions were concentrated and stored at − 80 °C after flash-freezing in liquid N_2_.

UV–visible absorption spectra were recorded at 10–20 µM protein concentration in a Cary Eclipse 50 spectrophotometer using 1 cm pathlength cuvettes, using an extinction coefficient of ε_280_ = 27,960 M^−1 ^cm^−1^ except for F25W for which we used ε_280_ = 33,460 M^−1^ cm^−1^.

### Circular dichroism (CD) spectroscopy

CD spectra were registered in a Jasco J-710 spectropolarimeter with a Peltier element for temperature control. Before the acquisition of the far-UV and near-UV/visible CD spectra, protein samples were buffer exchanged to 20 mM K-phosphate pH 7.4 using PD-10 columns (Cytiva, Barcelona, Spain). Spectra in the far-UV range were acquired using 6–7 µM of protein in a 1 mm path-length quartz cuvette. These spectra were registered in the 200–260 nm range, at 25 °C, with a scan rate of 100 nm min^−1^, a bandwidth of 1 nm and a time response of 2 s. Six spectra of each sample were acquired and averaged, and the corresponding blank (containing the same buffer but no protein) was acquired under the same conditions at the beginning and the end of each experimental series to assess for baseline drifts during the day, were averaged and subtracted from the spectra of protein samples. Similar conditions were used to acquire near-UV/visible CD spectra with some modifications. Protein concentration for these experiments was 15 µM and 10 spectra were acquired and averaged, the wavelength range was 270–330 nm and the path-length of the cuvette was 5 mm. To calculate mean molar residue ellipticities $\left[ \Theta \right]$_MRW_, Eq. (1) was used:

1 $$\left[ \Theta \right]_{MRW} = \frac{MRW \cdot \Theta }{{10 \cdot l \cdot c}}$$

where *MRW* is the molecular weight of each hPGK1 variant divided by 416 (the residues of native hPGK1 minus 1), $\Theta$ is the observed ellipticity of the sample in mdeg, *l* is the pathlength of the cuvette in cm and *c* the protein concentration in mg·mL^−1^.

### Thermal denaturation by CD spectroscopy

For thermal denaturation experiments by CD spectroscopy, we used exactly the same samples used to acquire CD wavelength-dependent spectra. Samples were thermostatized at 20 °C for 10 min and then temperature was raised to 75 °C at a 2 °C min^−1^ scan rate. Signals were acquired at 222 nm (for UV-CD in 1 mm path-length cuvettes) or 290 nm (for Near-UV CD in 5 mm path-length), using a bandwidth of 2 nm and a time response of 16 s. Raw data were normalized for linear pre- and post-transition baselines and the apparent *T*_m_ was determined as the temperature at which half of the *unfolded* fraction was observed by linear extrapolation. Thermal denaturation leads to irreversible protein aggregation (i.e. formation of protein aggregates of large size)^30^.

### Differential scanning calorimetry (DSC)

DSC analyses were carried out using a VP-DSC differential scanning calorimeter (Malvern Panalytical, Malvern, UK) with a cell volume of 137 µL. Experiments were performed in 20 mM HEPES–NaOH, 200 mM NaCl pH 7.4 and using 7–15 µM protein. Temperature range was 5–75 °C and scan rates were 1, 2.5 or 4 °C min^−1^. To investigate the effect of urea on hPGK1 denaturation, stock solutions of urea 5 M in 20 mM HEPES–NaOH, 200 mM NaCl pH 7.4 were diluted to the desired final concentration. All urea concentrations were checked by refractive index measurements.

The DSC traces were analysed using a two-state irreversible model^29,41^ (model 1), that describes very well the irreversible denaturation of hPGK1^29^. In this model, denaturation is considered as a kinetically-controlled conversion of the native (N) to a final state (F) that cannot fold back:

$$N\mathop{\longrightarrow}\limits^{\varvec {k} } F\quad ({\text{Model}}\;1)$$

This process is characterized by a strongly temperature-dependent first-order rate constant *k*. We also assume that the temperature dependence of *k* follows a slightly modified version of the Arrhenius equation (Eq. 2):

2 $$\ln k = \ln k_{T*} - \frac{{E_{a} }}{R} \left( {\frac{1}{T} - \frac{1}{T*}} \right)$$

where *T* is the absolute temperature, T* is a reference absolute temperature, *k*_T*_ is the rate constant at T^*^ and *E*_a_ is the activation energy and *R* is the ideal gas constant (1.987 cal mol^−1 ^K^−1^).

The fitting function used to extract relevant energetic parameters: *k*, *E*_a_, the enthalpy changes between N and F (Δ*H*) and the *T*_m_ (the maximum of the excess heat capacity versus *T*; usually close to the temperature at which* k* = 1 min^−1^) uses two linear pre- and post-transition temperature-dependent baselines smoothly connecting N and F signals. This procedure allow to *isolate* the calorimetric peak defined by the term $- \Delta H\cdot\left( {\frac{{dX_{N} }}{dT}} \right).$ This *peak* shows the dependence of the excess heat capacity *C*_p_^Exc^ from $\left( {\frac{{dX_{N} }}{dT}} \right)$ which is described by Eq. (3)^29,41,53^:

3 $$\frac{{dX_{N} }}{dT} = - \frac{{E_{a} }}{{RT_{m}^{2} }}\exp \left( {\frac{{E_{a} \cdot \Delta T}}{{RT_{m}^{2} }}} \right) \cdot \exp \left[ { - \exp \left( {\frac{{E_{a} \cdot \Delta T}}{{RT_{m}^{2} }}} \right)} \right]$$

From *C*_p_^Exc^ versus *T* profiles (i.e. after removing the chemical baselines), the value of the temperature-dependent rate constant *k* can be determined using Eq. (4)^54^:

4 $$k = \frac{{\nu \cdot C_{P}^{{Exc}} }}{{\Delta H - < H > }}$$

where *ν* is the scan rate in °C min^−1^, $C_{p}^{Exc}$ is the excess heat capacity at a given *T*, Δ*H* is enthalpy change of the transition and < *H*> is the excess enthalpy at a given *T*. This equation allows to determine the *T*-dependence of *k* and thus yields the corresponding Arrhenius plots (Eq. 2).

To determine kinetic *m* values (*m*^≠^) from DSC studies we followed the procedure described by^41^. Briefly, we carried out DSC experiments under the same conditions as described above but adding a final urea concentration in the range of 0.5–1.1 M. Then, *m*^≠^ values were determined using Eq. (5):

5 $$m^{ \ne } = - \frac{{E_{a} }}{{T_{m} }}\left( {\frac{{dT_{m} }}{{d\left[ {Urea} \right]}}} \right) - R \cdot T_{m} \cdot \left( {\frac{{d\ln \left( {\frac{{E_{a} }}{{RT_{m}^{2} }}} \right)}}{{d\left[ {Urea} \right]}}} \right)$$

where *E*_a_, *T*_m_, d*T*_m_/d[Urea] can be readily calculated from fittings at different urea concentrations. Thus, for each protein variant, three different values of *m*^≠^, one for each scan rate were obtained, and the data reported are the mean ± s.d. of these three values.

### Isothermal urea denaturation monitored by CD spectroscopy

Urea was prepared at ~ 6 M in 20 mM HEPES–NaOH, 200 mM NaCl, 1 mM 2-mercaptoethanol (β-ME), pH 7.4 and its actual concentration was determined by refractive index measurements. Urea stock solutions were mixed with hPGK1 solutions in 20 mM HEPES–NaOH, 200 mM NaCl, 1 mM β-ME, pH 7.4 to a final concentration of 5 μM (in protein) and 0–5 M (in urea). Samples were incubated for at least 2 h and denaturation experiments were monitored by Far-UV CD spectroscopy similarly to those described in Section "Circular dichroism (CD) spectroscopy". but restricting the acquisition range to 215–260 nm.

Data at 222 nm were used for fittings assuming a two-state model (apparently valid for WT hPGK1 using different conformational probes) ^3,28–30^ to provide the values of experimental equilibrium *m* (*m*_urea_) and *C*_m_ (concentration of urea at which half-denaturation occurs) using Eq. (6):

6 $$S = \left[ {S_{N} + m_{N} \cdot \left[ {urea} \right] + \left( {S_{U} + m_{U} } \right) \cdot \left( {\exp^{{\frac{{m_{urea} \cdot \left( {\left[ {urea} \right] - C_{m} } \right)}}{R \cdot T}}} } \right)} \right]/\left[ {1 + \left( {\exp^{{\frac{{m_{urea} \cdot \left( {\left[ {urea} \right] - C_{m} } \right)}}{R \cdot T}}} } \right)} \right]$$

where *S* is the experimental CD signal as a function of the [urea], *S*_N_ and *S*_U_ are the fitted CD signals for the Native and Unfolded state baselines at 0 M urea, respectively, *m*_N_ and *m*_U_ are the slopes of the native and unfolded state baselines, *m*_urea_ describes the unfolding cooperativity, *R* is the ideal gas constant and *T* is the experimental temperature (298.15 K). Although this model is likely an oversimplification, it provides a proxy to compare the cooperativity of reversible chemical unfolding of hPGK1 variants.

### Proteolysis by thermolysin

Thermolysin from *Geobacillus stearothermophilus* (Sigma-Aldrich, Madrid, Spain) was prepared in 20 mM HEPES–NaOH, 200 mM NaCl, 100 mM CaCl_2_, pH 7.4 and its concentration was determined spectrophotometrically using ε_280_ = 66,086 M^−1^ · cm^−1^ based on the protein primary sequence. Proteolysis was initiated after 5 min incubation at 25 °C by mixing hPGK1 variants and thermolysin to a final concentrations of 10 µM and 0.5 or 1 µM, respectively. When the initial reaction was started contained 10 mM CaCl_2_. Aliquots were withdrawn at different times (2 min–96 h), mixed with 25 mM EDTA (final concentration), pH 8 and denatured with Laemmli´s buffer (1:1 in V/V) at 95 °C for 5 min. Samples were resolved in SDS-PAGE gels (12% acrylamide) and analysed by densitometry using the software ImageJ (https://imagej.nih.gov/ij/).

The decay of the full-length protein (*I*) versus time (*t*) was fitted using the following single exponential function to provide the first-order kinetic constant *k*_*obs*_:

$$I = I_{0} \cdot \exp^{{ - k_{obs} \cdot t}}$$

The half-life (t_1/2_) for proteolysis was determined using: *t*_1/2_ = ln 2/*k*_obs_. Errors corresponded to those from the fittings.

### Hydrogen/deuterium eXchange mass spectrometry (HDX-MS)

Amide hydrogen/deuterium exchange (HDX) of hPGK1 was studied for the WT and mutant variants F25A, F25G, F25L, F25V, F25W as described previously^55^ with some modifications. Briefly, 20 μM of protein solution was 10 × diluted with a D_2_O-based 20 mM HEPES–NaOH, 200 mM NaCl, 0.5 mM TCEP (tris(2-carboxyethyl)phosphine), pD 7.4, to start the exchange reaction. The exchange was terminated after 10 s, 30 s, 2 min, 5 min, 20 min, 1 h, 3 h 20 min and 20 h by mixing (1:1) with 0.5 M Glycine–HCl, pH 2.3 and the samples were flash frozen in liquid N_2_. Time points 30 s, 5 min and 3 h 20 min were replicated. Custom-made nepenthesin-2 column was used for online proteolysis and the peptides were trapped and desalted on a SecurityGuard™ pre-column (ULTRA Cartridges UHPLC Fully Porous Polar C18, 2.1 mm, Phenomenex, Torrance, CA, USA). Solvent for this step—0.4% formic acid (FA) in water—was pumped by a 1260 Infinity II Quaternary pump (Agilent Technologies, Waldbronn, Germany) at a flow rate of 150 μl min^−1^. Subsequent peptide separation was performed on an analytical column (LUNA^®^ Omega Polar C18 Column, 100 Å, 1.6 µm, 100 mm × 1.0 mm, Phenomenex, Torrance, CA, USA) using a linear gradient (5–45% B in 6 min) followed by a quick step to 99% B lasting 5 min and pumped by 1290 Infinity II LC System (Agilent Technologies, Waldbronn, Germany) at a flow rate of 40 μl min^−1^. Solvent A was 0.1% FA/2% acetonitrile (ACN) in water, B was 0.1% FA/98% ACN in water. Digestion, desalting, and separation were performed at 0 °C and pH 2.3 to minimize deuterium back-exchange. LC system was directly coupled to an ESI source of 15 T FT-ICR mass spectrometer (solariX XR, Bruker Daltonics, Bremen, Germany) operating in broad-band MS mode. A fully deuterated control was prepared for each hPGK1 variant and was used to correct back-exchange levels as described previously^55,56^. MS data were exported using DataAnalysis 5.3 and processed by in-house developed program Deutex^57^. Peptide identification was done in a separate data-dependent LC–MS/MS run performed using the identical LC settings but connected to ESI-timsTOF Pro with PASEF. LC–MS/MS data were exported to mgf files using DataAnalysis 5.3 and searched by MASCOT (Matrix Science, London, UK) against a database containing hPGK1 sequences, nepenthesin-2 and cRAP contaminant sequences. Decoy search was enabled with a false-discovery ratio < 1% and an Ion Score cut-off 15.

### Computational analyses of the F25 mutational effects

The Wako-Saitô-Muñoz-Eaton (WSME) model based perturbation analysis of hPGK1 was performed as discussed before for mutants of the protein NQO1^58^. Briefly, an ensemble of 8,398,795 microstates is constructed employing the block-version of the WSME model—bWSME with a most probable block length of 5 consecutive residues—and the PDB structure 2XE7^59,60^. The microstate energetics include stabilizing contributions from van der Waals interactions (heavy-atom interactions identified with a 5 Å cut-off with a mean-field interaction energy of -78 J mol^−1^ per heavy atom contact), all-to-all electrostatics at pH 7 protonation state, and simplified solvation free energy (defined by the heat capacity change of − 0.36 J mol^−1^ K^−1^ per native contact). In addition, an entropic penalty of − 14.5 J mol^−1^ K^−1^ per residue is introduced when fixing an unfolded residue to a folded conformation. The balance between the stabilization (free-)energy terms and entropic penalty determines the free energy of every microstate, the statistical weight and hence the associated probability^59^.

## Supplementary Information

Supplementary Information.

## Data Availability

The datasets generated and/or analyzed during the current study are available from the corresponding author on reasonable request (A.L.P.).

## Abbreviations

BB: Binding buffer
CD: Circular dichroism
CTD: C-terminal domain
DSC: Differential scanning calorimetry
*E*_a_: Activation energy
HDX-MS: Hydrogen/deuterium exchange monitored by mass spectrometry
hPGK1: Human phosphoglycerate kinase, isoform 1
IMAC: Immobilized-metal affinity chromatography
LB: Luria–Bertani
NTD: N-terminal domain
PGK: Phosphoglycerate kinase
SEC: Size-exclusion chromatography
SDS-PAGE: Poly-acrylamide gel electrophoresis in the presence of sodium dodecyl-sulphate
WSME model: Wako–Saitô–Muñoz–Eaton model
WT: Wild-type
β-ME: 2-Mercaptoethanol
% D: Percentage of deuterium incorporation
