# Supplementary material for: Loss of stability and unfolding cooperativity in hPGK1 upon gradual structural perturbation of its N-terminal domain hydrophobic core
Source: Sci Rep. 2022 Oct 13;12:17200. doi: 10.1038/s41598-022-22088-1 (PMC9561527; doi:10.1038/s41598-022-22088-1)
Supplement: Supplementary file 1 — Supplementary Information. [file 41598_2022_22088_MOESM1_ESM.docx]

**Loss of stability and unfolding cooperativity in hPGK1 upon gradual structural perturbation of its N-terminal domain hydrophobic core**

**Juan Luis Pacheco-García, Dmitry S. Loginov, Athi N. Naganathan, Pavla Vankova, Mario Cano-Muñoz, Petr Man and Angel L. Pey.**

#### Supplementary Material

**Table S1. Crystal structures of hPGK1 in the presence of different ligands.** All data were retrieved from the PDB site (accessed February 24^th^, 2022; <https://www.rcsb.org/>). The % of accessible surface area (%ASA) was calculated for F25 in all these structures using GetArea (<http://curie.utmb.edu/getarea.html>) ^1^.

| **PDB Code** | **Ligands** | **% ASA F25** | **Reference** |
| --- | --- | --- | --- |
| 3C3A | L-ADP, 3-PG and Mg^2+^ | 0.0 | ^2^ |
| **3C3C** | L-CDP, 3-PG and Mg ^2+^ | 0.0 | ^2^ |
| **3C39** | 3-PG | 0.0 | ^2^ |
| **3C3B** | L-CPD, PO_4_^2-^ | 0.0 | ^2^ |
| **2ZGV** | D-ADP | 0.0 | ^2^ |
| **2YBE** | 3-PG, ALF_4_^-^, Cl^-^, L-ADP, Mg^2+^ | 0.0 | ^2^ |
| **2Y3I** | 3-PG, AlF_4_^-^, Cl^-^, L-ADP, Mg^2+^ | 0.0 | ^3^ |
| **2X15** | 3-PG, 1,3-BPG, L-ATP, L-ADP, Mg^2+^ | 0.0 | Bowler, M.W., Cliff, M.J., Marston, J.P.M., Baxter, N.J., Hounslow, A.M.H., Varga, A.V., Szabo, J., Vas, M., Blackburn, G.M., Waltho, J.P.  To be published |
| **3ZOZ** | L-ADP, 3-PG, MgF_3_^-^, Br^-^ | 0.0 | Bowler, M.W.  To be published |
| **2WZC** | L-ADP, 3-PG, Mg^2+^, Cl^-^, AlF_4_^-^ | 0.0 | ^4^ |
| **2X13** | L-ADP, 3-PG and Mg^2+^ | 0.0 | Bowler, M.W., Cliff, M.J., Marston, J.P.M., Baxter, N.J., Hownslow, A.M.H., Varga, A.V., Szabo, J., Vas, M., Blackburn, G.M., Waltho, J.P.  To be published. |
| **2WZB** | L-ADP, 3-PG, MgF_3_^-^, Mg^2+^, Cl^-^ | 0.0 | ^4^ |
| **2XE7** | L-ADP, 3-PG | 0.0 | ^5^ |
| **2XE6** | 3-PG | 0.0 | ^5^ |
| **2XE8** | AMP-PNP, 3-PG | 0.0 | ^5^ |
| **4AXX** | L-ADP, 3-PG, BeF_3_, Mg^2+^, Cl^-^ | 0.0 | Bowler, M.W., Cliff, M.J., Blackburn, G.M., Waltho, J.P.  To be published. |

**Table S2. Naturally-occurring mutations that cause a decrease (*Cavity* set) or an increase (*Strain* set) in the size-chain or affect glycine residues (*Gly* set) by removing or introducing Gly.** Strain or Cavity mutations lead to a change in volume of at least ± 15% based on the average values reported by ^6^. In these three sets, mutations that affect charge (E, D, K or R), H and P residues were not included. In the Strain and Cavity sets, mutations involving Gly residues were not included. Changes in side-chain polarity were allowed. Underlined mutations are those affecting buried residues (< 10% solvent accessibility) in the hPGK1 structure (PDB 2XE7) and using the GetArea online server. Mutations were retrieved from the ClinVar (72 mutations)(https://www.ncbi.nlm.nih.gov/clinvar/) and COSMIC (99 mutations)(https://cancer.sanger.ac.uk/cosmic) databases, both accessed by 13^th^ September 2022.

| **ClinVar database** | |
| --- | --- |
| ***Set*** | ***Mutations*** |
| *Cavity* | **I34V**, **I47N**, V83A, **M176V**, N180S, **F244V**, **I253T**, T298A, **F348C**, T363S, **I371V**. |
| *Strain* | S4F, **C50W**, **S62N**, **V81F**, A107V, A109V, **V266M**, **V278M**, L282F, **A296V**, **S320N**, A354T, **A398T**. |
| *Gly* | **C50G**, **G158V**, G254A, **G306V**, W311G, **G372C**, **G372S**, S415G. |
| **COSMIC database** | |
| ***Set*** | ***Mutations*** |
| *Cavity* | N53S, **F95L**, **L122I**, N195S, **F197I**, **L414V**. |
| *Strain* | T8M, **V14I**, **V20I**, **S46N**, **V104L**, S115T, S136Y, S136F, A152S, A198T, **A200V**, S364Y, A377S, A377V, A381T, T385M, **T393I**. |
| *Gly* | G130W, **G158V**. |

**Figure S1. Thermal stability of WT, F25L and F25V determined by DSC in the presence of different urea concentrations.** Each panel shows the experimental data for a given variant, urea concentration and at different scan rates. Lines are fits to a two-state irreversible model ^7^.

**
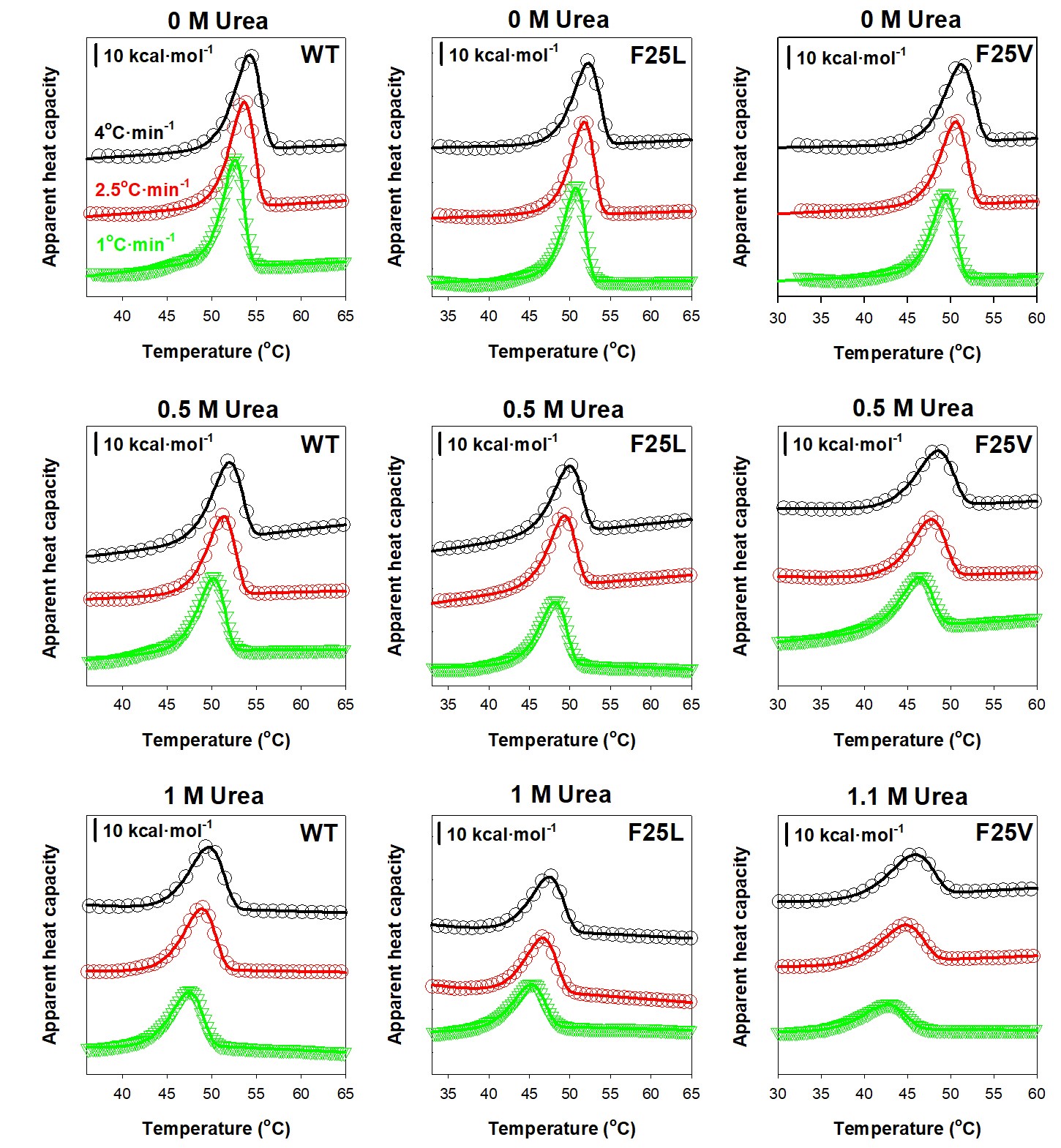
**

**Figure S2. Thermal stability of F25A, F25G and F25W determined by DSC in the presence of different urea concentrations.** Each panel shows the experimental data for a given variant, urea concentration and at different scan rates (color code for scan rates is the same that in Figure S1). Lines are fits to a two-state irreversible model ^7^.


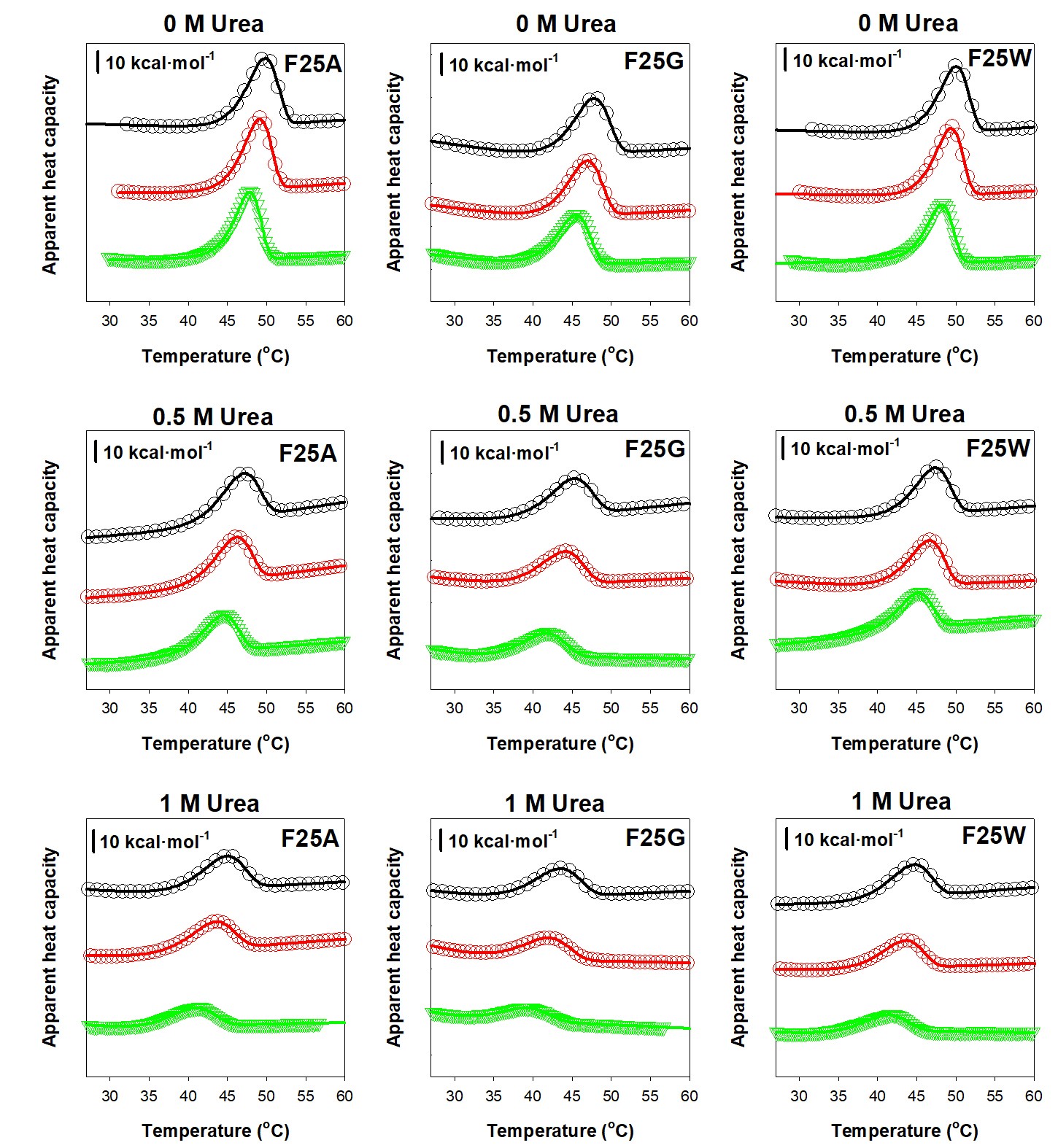


**Figure S3. Determination of *m*^≠^ values for hPGK1 variants using DSC and based on equation 5**. The value of left-side of equation 5 was calculated for each variants from left panels. The right-side of equation 5 was calculated from the right panels. All the calculations were made according to ^7^.


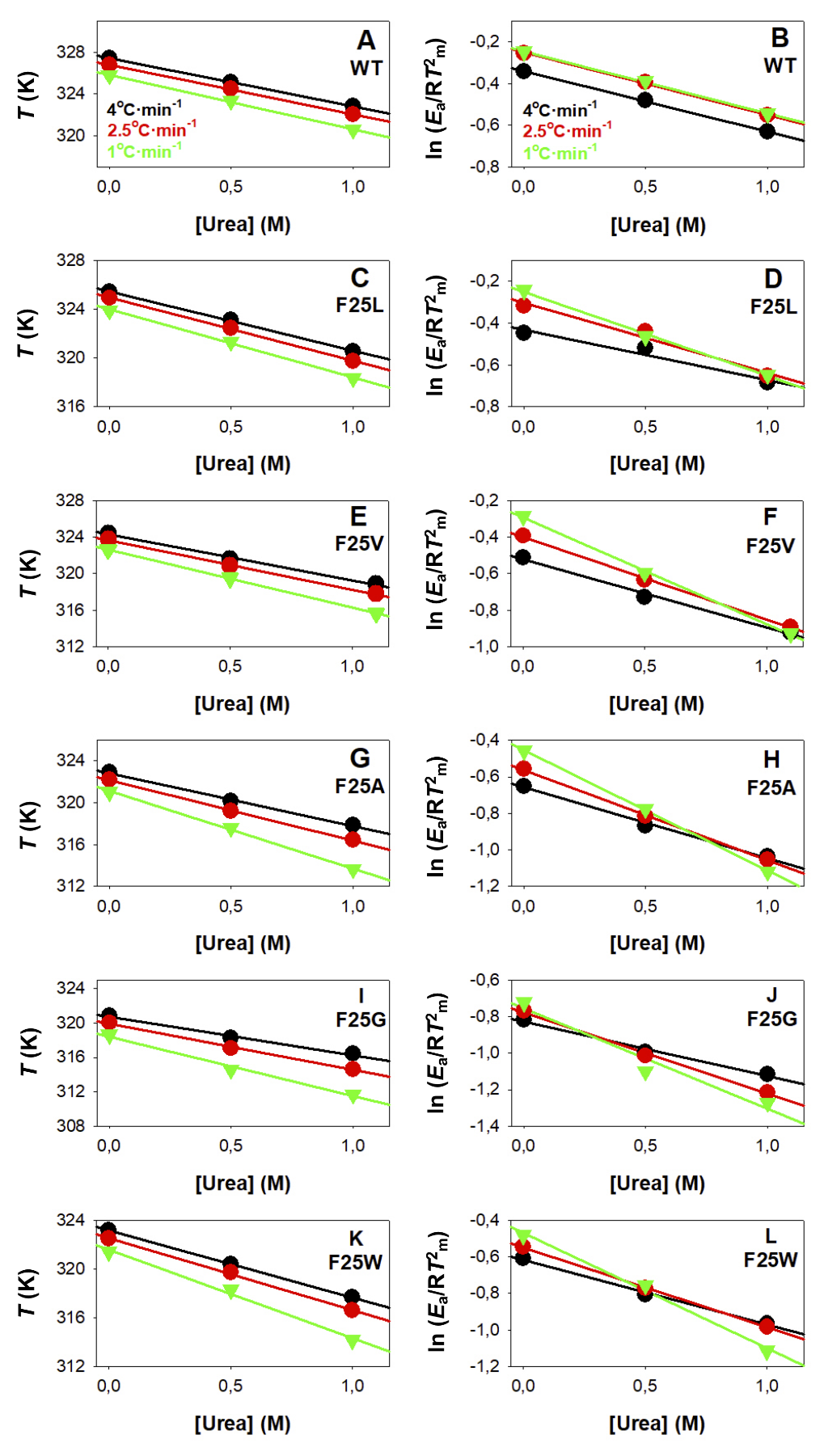


**Figure S4. Conformational stability of F25 mutants as determined by proteolysis with thermolysin.** A) Representative SDS-PAGE gels of hPGK1 variants proteolysis kinetics; B) Densitometric analysis of results shown in panel A with fittings to a single exponential function (left panel) and the corresponding half-lives (right panel). Thermolysin concentration was 0.5 µM. Temperature was 25^o^C.


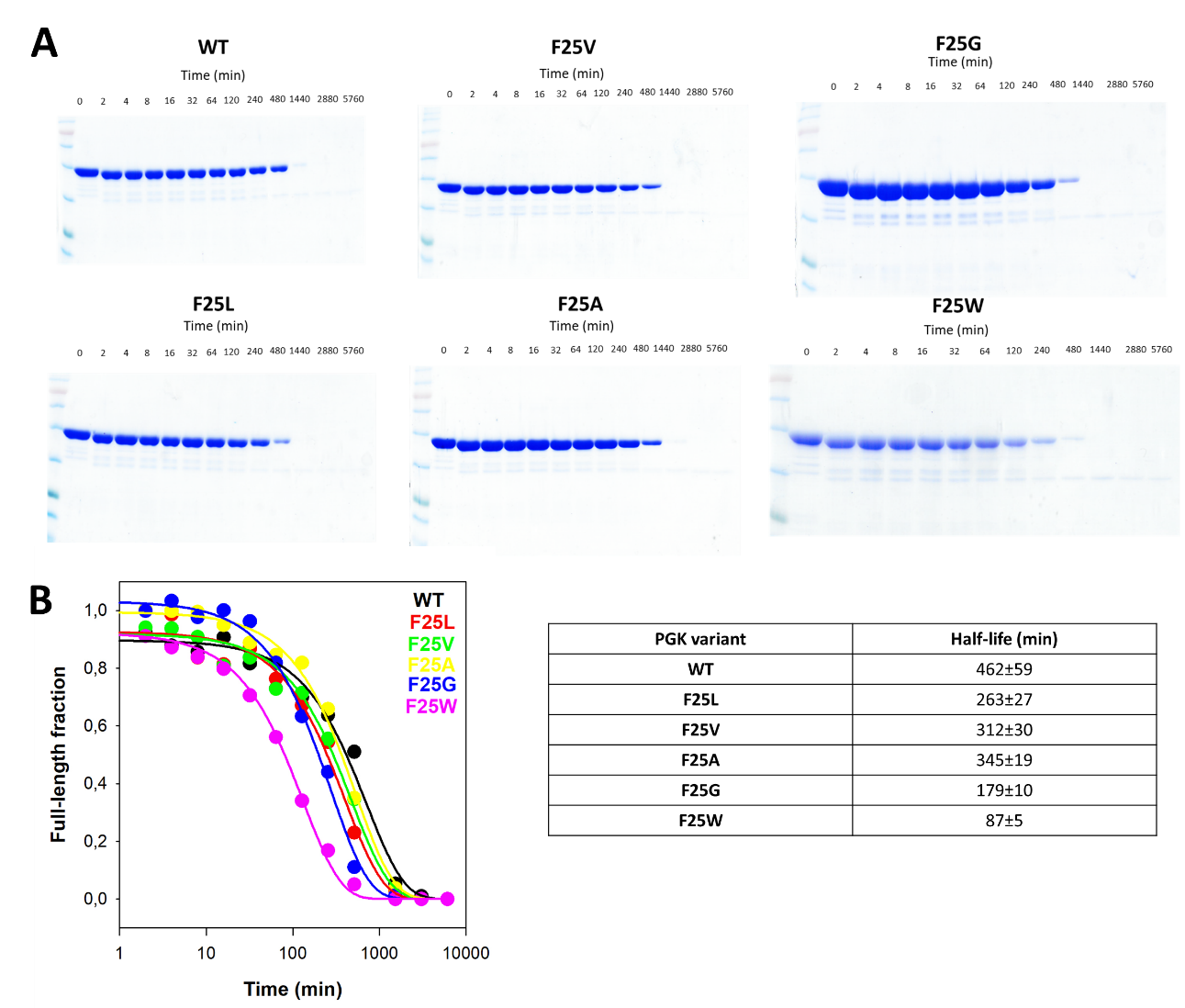


**Figure S5. Kinetics of D incorporation to hPGK1 F25L.** A) % of D incorporation at different time points for the entire hPGK1 protein. Note that segments 13-17, 265-269 and 345-347 were not detected. B) The value of Δ%D_av_ for the F25L variant was calculated using the WT protein as reference. C) Selected transients for segments displaying faster kinetics (Δ%D_av_ > 10 %). Lines generally show best-fits to a double-exponential function including a burst phase.

**
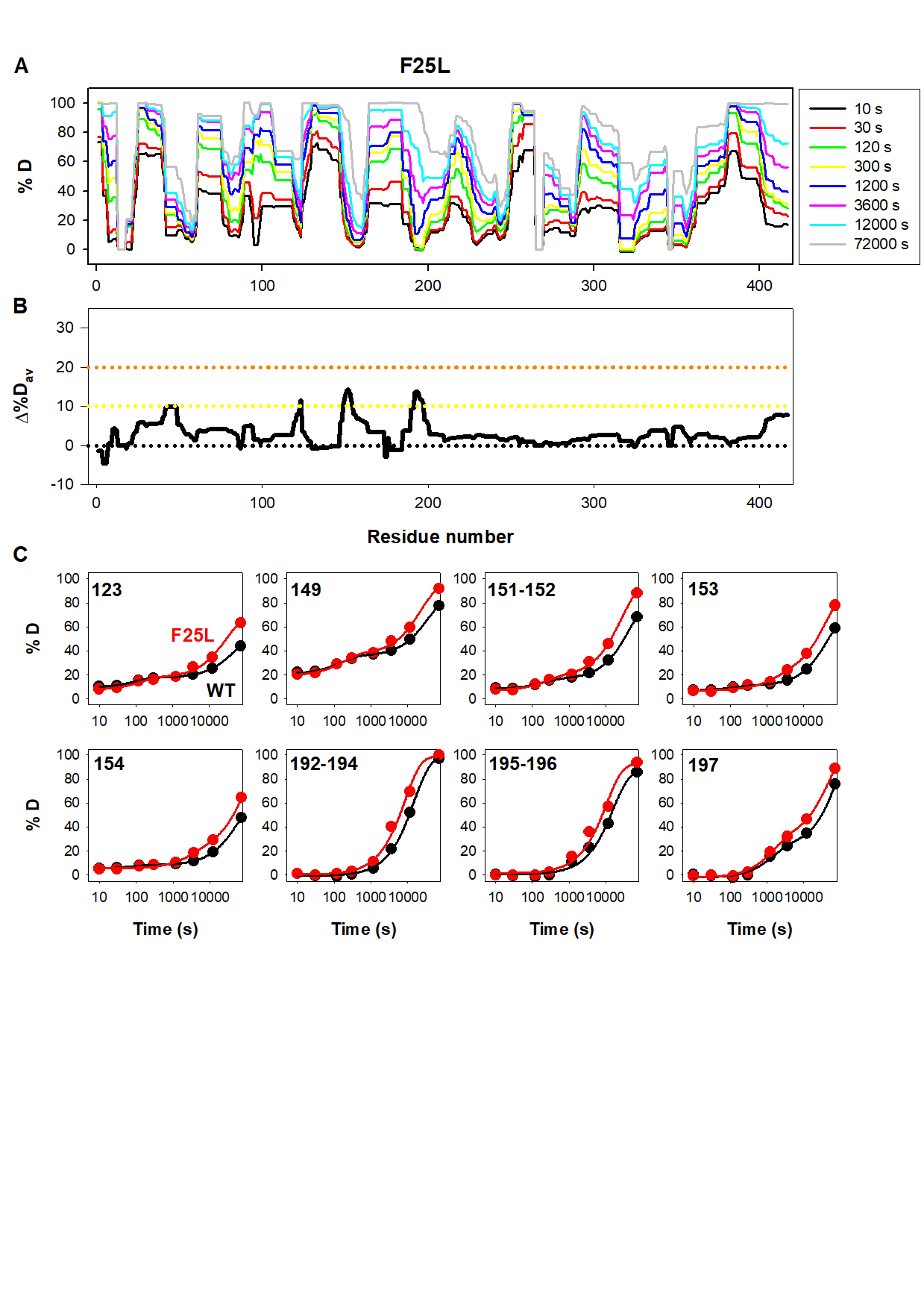
**

**Figure S6. Kinetics of D incorporation to hPGK1 F25V.** A) % of D incorporation at different time points for the entire hPGK1 protein. Note that segments 13-17, 265-269 and 345-347 were not detected. B) The value of Δ%D_av_ for the F25V variant was calculated using the WT protein as reference. C) Selected transients for segments displaying faster kinetics (Δ%D_av_ > 10 %). Lines generally show best-fits to a double-exponential function including a burst phase.

**Figure S7. Kinetics of D incorporation to hPGK1 F25A.** A) % of D incorporation at different time points for the entire hPGK1 protein. Note that segments 13-17, 265-269 and 345-347 were not detected. B) The value of Δ%D_av_ for the F25A variant was calculated using the WT protein as reference. C) Selected transients for segments displaying faster kinetics (Δ%D_av_ > 10 %). Lines generally show best-fits to a double-exponential function including a burst phase.


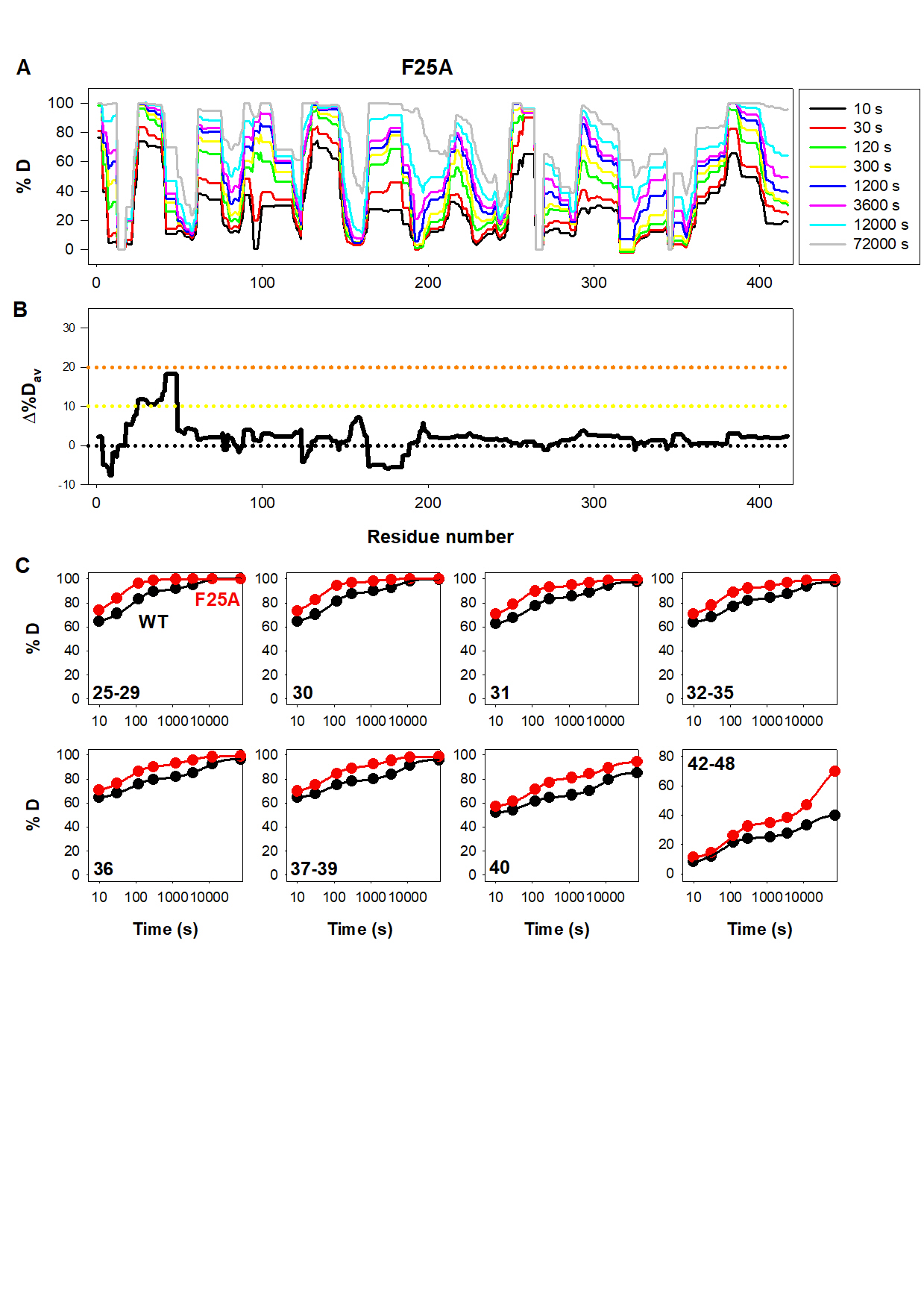


**Figure S8. Kinetics of D incorporation to hPGK1 F25G.** A) % of D incorporation at different time points for the entire hPGK1 protein. Note that segments 13-17, 265-269 and 345-347 were not detected. B) The value of Δ%D_av_ for the F25G variant was calculated using the WT protein as reference. C) Selected transients for segments displaying faster kinetics (Δ%D_av_ > 10 %). Lines generally show best-fits to a double-exponential function including a burst phase.


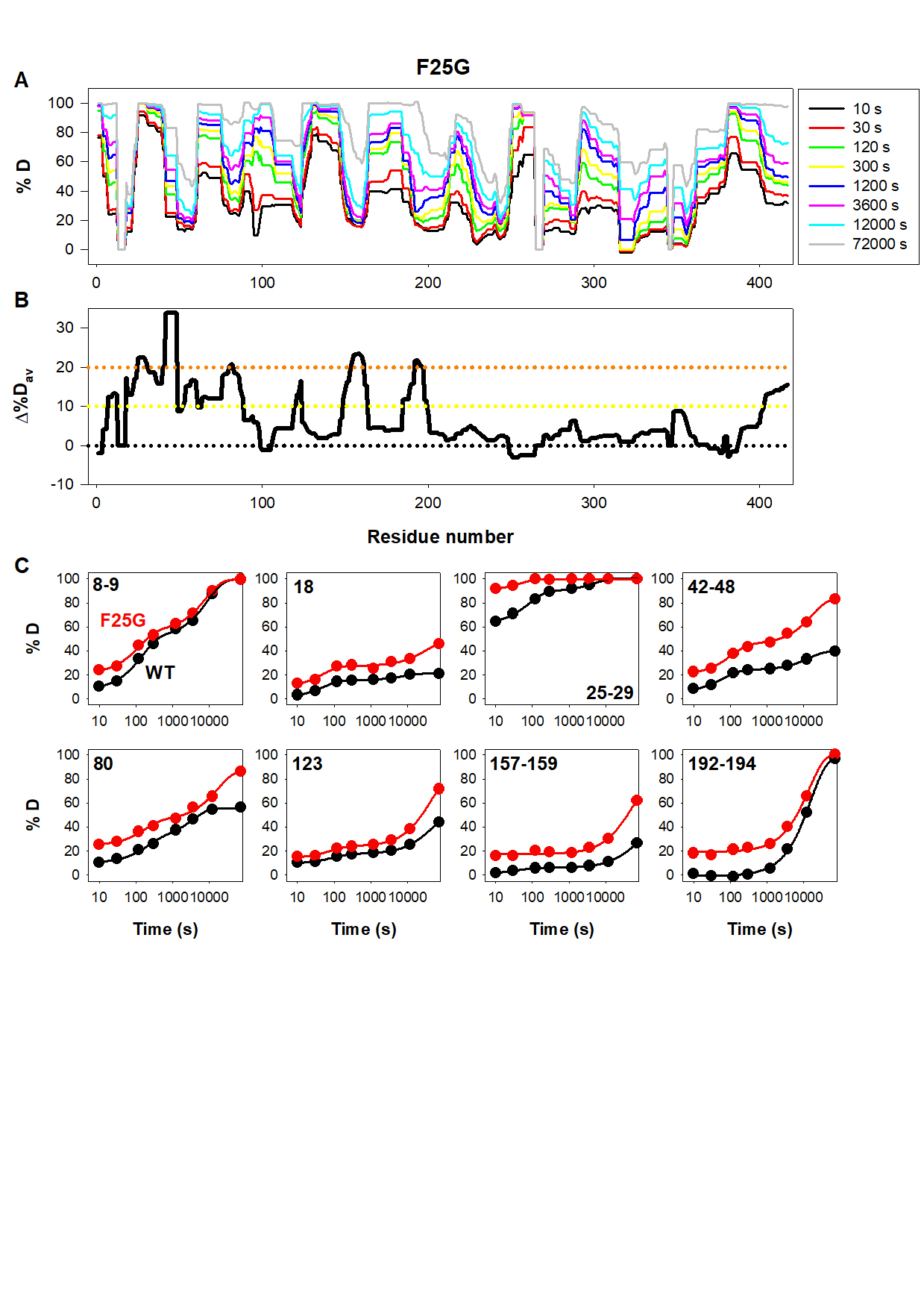


**Figure S9. Kinetics of D incorporation to hPGK1 F25W.** A) % of D incorporation at different time points for the entire hPGK1 protein. Note that segments 13-17, 265-269 and 345-347 were not detected. B) The value of Δ%D_av_ for the F25W variant was calculated using the WT protein as reference. C) Selected transients for segments displaying faster kinetics (Δ%D_av_ > 10 %). Lines generally show best-fits to a double-exponential function including a burst phase.


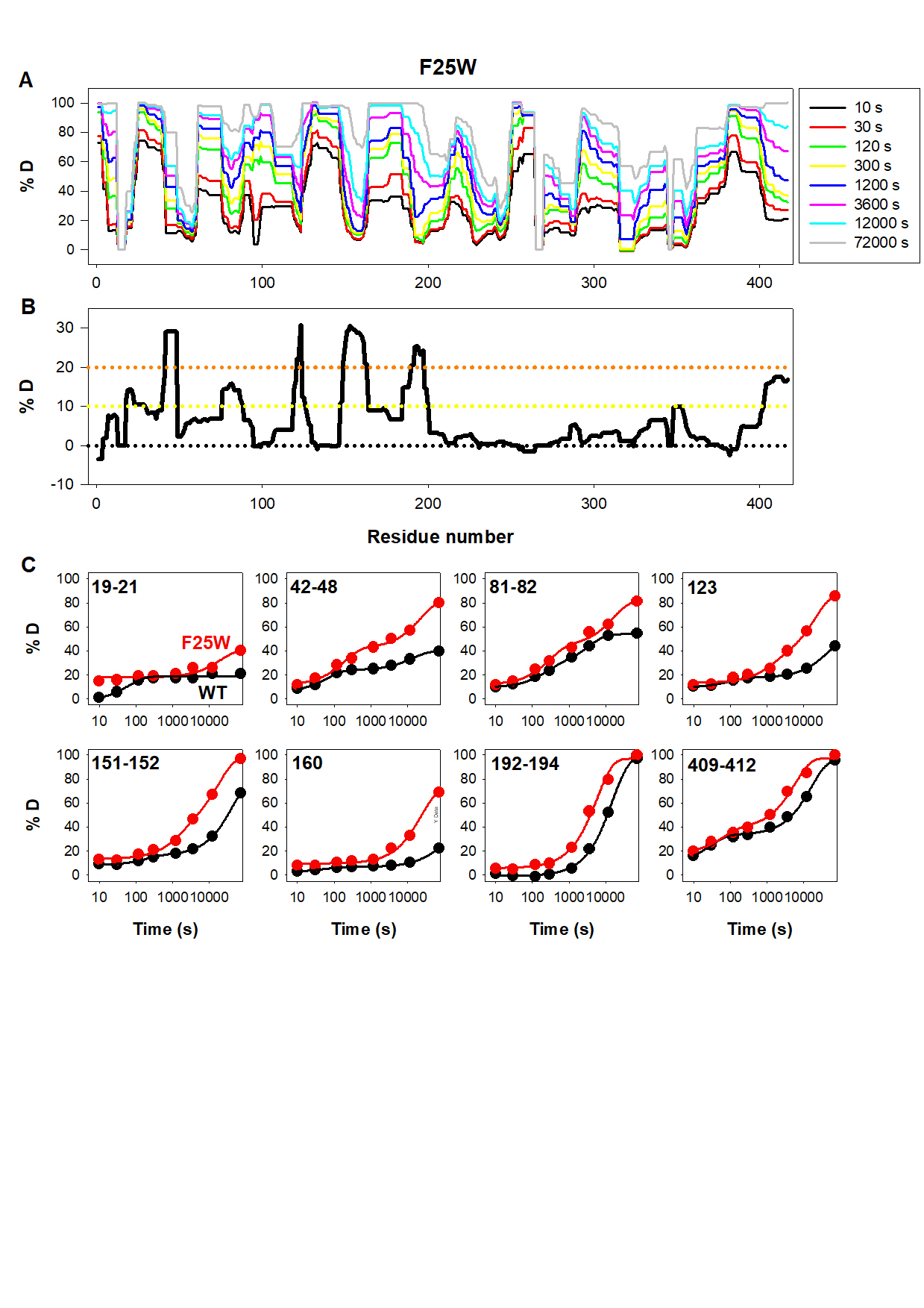


**Figure S10. NTD:CTD interaction surface in hPGK1.** A) Representation of the NTD (1, shown in ribbons and colored in red) and the CTD (2, shown in ribbons and colored in blue). B and C) Molecular surface of amino acids from each domain participating in the interdomain interface (in translucid red and blue, respectively, in B and C). The surface area of interaction in each case is shown in the Figure. The open conformation of hPGK1 (PDB ID: 2XE7)^5^ was used for calculations. Ligands were removed and missing amino acids were added using YASARA Structure (v.17.12.24) molecular modeling software ^8^. To remove bumps and correct the covalent geometry, the structure was energy-minimized with the AMBER14 force field ^9^, using a 8 Å force cutoff and the Particle Mesh Ewald algorithm ^10^ to treat long range electrostatic interactions. After removal of conformational stress by a short steepest descent minimization, the procedure continued by simulated annealing Simulated annealing minimizations started at 298K, atom velocities were scaled down by 0.9 every ten steps for a total time of five ps with a timestep of 2 fs, until convergence was reached, i.e. the energy improved by less than 0.05 kJ/mol per atom during 200 steps. Simulations were performed using YASARA Structure with explicit solvent (TIP3P water, the solvent density was equilibrated to a final value of 0.997 g/mL) in a periodic box with size 10 Å larger than the protein in every dimension. Ewald summation was used to assign amino acid charge according to their predicted side chain pK_a_ and was neutralized by adding counterions (NaCl). The Cα-RMSD retrieved for the structure after simulation was 0.153 Å. Residues forming the interaction interface from each domain were calculated with the ListRes command in YASARA software with a cutoff distance of 5 Å.


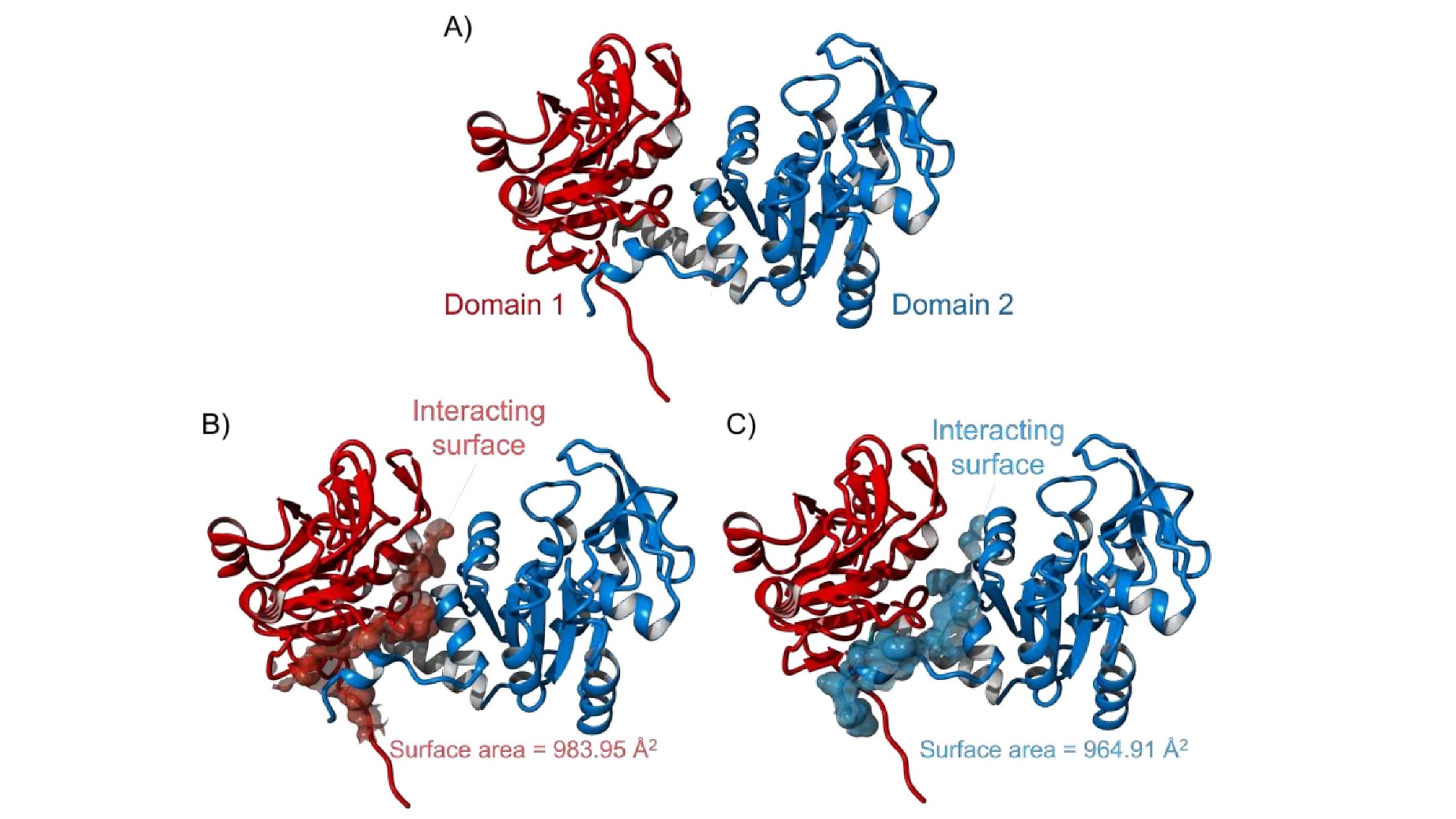


**Figure S11. Destabilization of the NTD:CTD interaction surface by the F25 mutations.** A) Percentage of residues affected by F25 mutations in the interaction surface based on HDX-MS (with at least a 10% increase in Δ%D_av_). B) Degree of destabilization of the residues belonging to interaction surface. Data for individual residues are displayed as black circles, whereas the average effect (±s.d.) is shown as cyan squares.


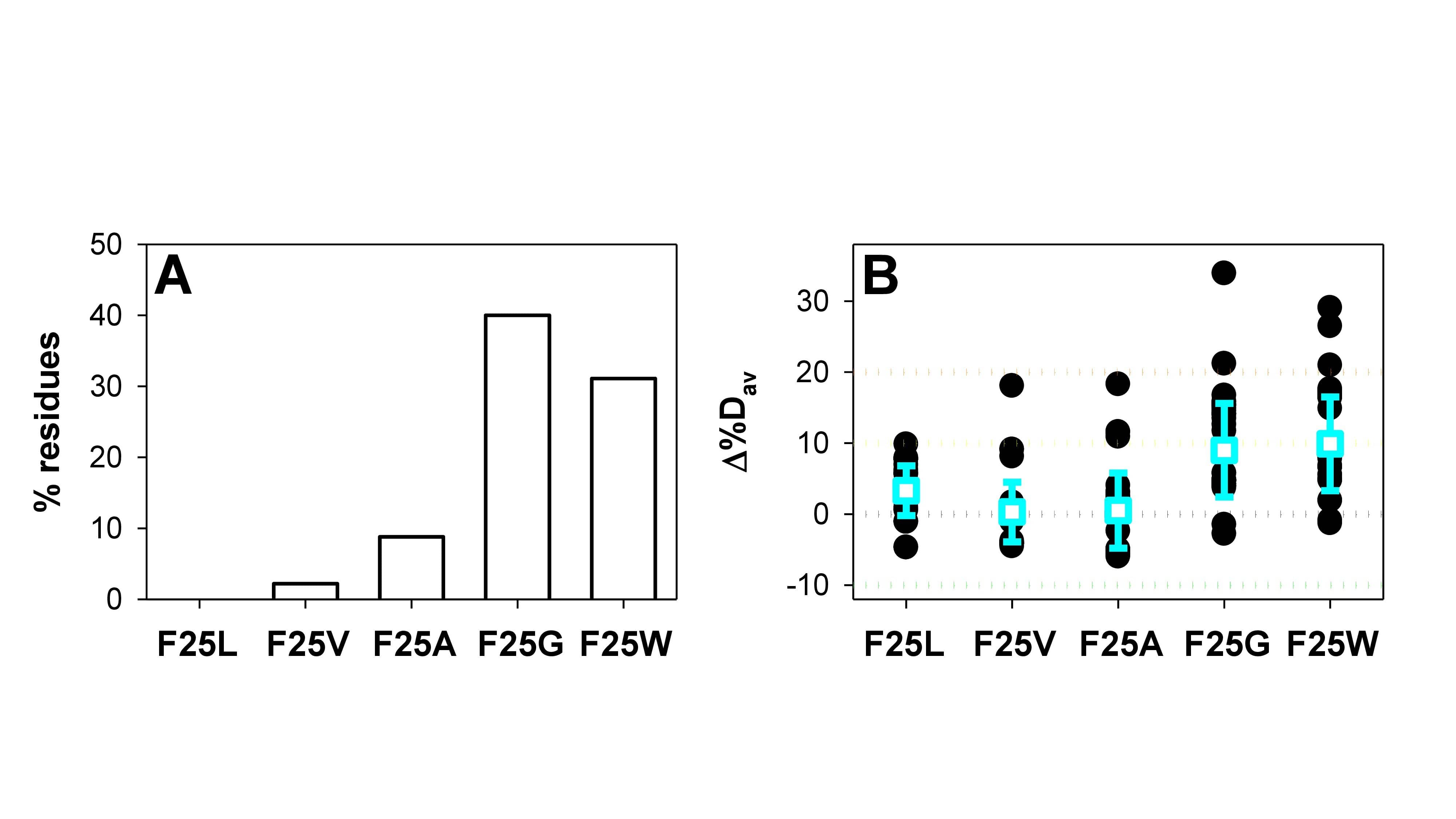


**Supplementary references**

1. Fraczkiewicz, R. & Braun, W. Exact and efficient analytical calculation of the accessible surface areas and their gradients for macromolecules. *J Comput Chem* **19**, 319-333 (1998).

2. Gondeau, C. *et al.* Molecular basis for the lack of enantioselectivity of human 3-phosphoglycerate kinase. *Nucleic Acids Res* **36**, 3620–9 (2008).

3. Lallemand, P. *et al.* Interaction of human 3-phosphoglycerate kinase with its two substrates: is substrate antagonism a kinetic advantage? *J Mol Biol* **409**, 742–57 (2011).

4. Cliff, M. J. *et al.* Transition state analogue structures of human phosphoglycerate kinase establish the importance of charge balance in catalysis. *J Am Chem Soc* **132**, 6507–16 (2010).

5. Zerrad, L. *et al.* A Spring-loaded Release Mechanism Regulates Domain Movement and Catalysis in Phosphoglycerate Kinase. *Journal of Biological Chemistry* **286**, 14040–14048 (2011).

6. Counterman, A. E. & Clemmer, D. E. Volumes of Individual Amino Acid Residues in Gas-Phase Peptide Ions. *J Am Chem Soc* **121**, 4031–4039 (1999).

7. Rodriguez-Larrea, D., Minning, S., Borchert, T. v & Sanchez-Ruiz, J. M. Role of solvation barriers in protein kinetic stability. *J Mol Biol* **360**, 715–24 (2006).

8. Krieger, E. & Vriend, G. YASARA View - molecular graphics for all devices - from smartphones to workstations. *Bioinformatics* **30**, 2981–2 (2014).

9. Hornak, V. *et al.* Comparison of multiple Amber force fields and development of improved protein backbone parameters. *Proteins* **65**, 712–25 (2006).

10. Essmann, U. *et al.* A smooth particle mesh Ewald method. *J Chem Phys* **103**, 8577–8593 (1995).
